# Transmissible microbial and metabolomic remodeling by soluble dietary fiber improves metabolic homeostasis

**DOI:** 10.1038/srep10604

**Published:** 2015-06-04

**Authors:** Baokun He, Kazunari Nohara, Nadim J. Ajami, Ryan D. Michalek, Xiangjun Tian, Matthew Wong, Susan H. Losee-Olson, Joseph F. Petrosino, Seung-Hee Yoo, Kazuhiro Shimomura, Zheng Chen

**Affiliations:** 1 Department of Biochemistry and Molecular Biology, The University of Texas Health Science Center at Houston, 6431 Fannin St., Houston, TX 77030; 2 The Alkek Center for Metagenomics and Microbiome Research, Department of Molecular Virology and Microbiology, One Baylor Plaza, Baylor College of Medicine, Houston, TX 77030; 3 Metabolon Inc., 617 Davis Drive, Durham, NC 27713; 4 RhythMed, 14673 W Birch Lane, Wadsworth, IL 60083; 5 Matsutani America Inc., 500 Park Blvd. Suite 1240, Itasca, IL 60143

## Abstract

Dietary fibers are increasingly appreciated as beneficial nutritional components. However, a requisite role of gut microbiota in fiber function and the overall impact of fibers on metabolomic flux remain unclear. We herein showed enhancing effects of a soluble resistant maltodextrin (RM) on glucose homeostasis in mouse metabolic disease models. Remarkably, fecal microbiota transplantation (FMT) caused pronounced and time-dependent improvement in glucose tolerance in RM recipient mice, indicating a causal relationship between microbial remodeling and metabolic efficacy. Microbial 16S sequencing revealed transmissible taxonomic changes correlated with improved metabolism, notably enrichment of probiotics and reduction of *Alistipes* and *Bacteroides* known to associate with high fat/protein diets. Metabolomic profiling further illustrated broad changes, including enrichment of phenylpropionates and decreases in key intermediates of glucose utilization, cholesterol biosynthesis and amino acid fermentation. These studies elucidate beneficial roles of RM-dependent microbial remodeling in metabolic homeostasis, and showcase prevalent health-promoting potentials of dietary fibers.

Dietary management and intervention is increasingly appreciated as a vital strategy to combat the worldwide epidemic of metabolic syndrome. One important class of beneficial food components is dietary fibers, known as plant-derived complex polysaccharides resistant to digestion by amylases and glycoamylases in the small intestine^1^,^2^,^3^. Whereas insoluble fibers promote colonic regularity and gastrointestinal (GI) function mainly through physical bulking effects, soluble dietary fibers, such as inulin, oligofructosaccharide, and resistant maltodextrin, have shown diverse health benefits both locally in the gastrointestinal (GI) tract and systemically throughout the body^1^,^4^,^5^. In particular, a large body of studies using both animal models and human subjects highlight an important role of fibers in energy metabolism, serving to blunt body weight gain and improve glucose and lipid homeostasis^6^,^7^,^8^. Detailed knowledge of the underlying functional mechanisms is thus important to fully exploit the health benefits of dietary fibers.

Accumulating evidence underscores a functional relationship between soluble dietary fibers and gut microbiota in the large intestine^9^,^10^,^11^. Soluble fibers are recognized prebiotics able to enrich probiotic bacteria, most notably *Lactobacillus* and *Bifidobacterium*, that are beneficial for digestive function, mucosal integrity and immune response^12^,^13^,^14^. On the other hand, dietary fibers are fermented by gut bacteria, producing metabolites for energy and signaling needs^15^,^16^. The primary fermentation products of dietary fibers are short-chain fatty acids (SCFAs), mainly produced by the predominant phyla *Firmicutes* and *Bacteriodetes*^3^,^17^,^18^. SCFAs can provide energy for distinct tissues^13^,^19^, and several recent studies have also revealed novel mechanisms whereby SCFAs act on membrane receptors and nutrient sensors to regulate physiological processes including glucose homeostasis^8^,^20^,^21^,^22^. Together, these studies highlight important roles of gut microbiota in the metabolic regulation by fibers.

A number of fundamental questions remain concerning the functional relationship between microbiota and fiber. First, although microbiome profiling has revealed extensive correlation between fiber intake and microbial shift, a requisite role of gut microbiota for metabolic regulation by fibers has not been unequivocally established^16^. Second, whereas SCFAs are well established metabolites of fiber, global changes in host metabolic network remains poorly understood, thus hampering identification of additional key metabolic pathways and in-depth mechanistic understanding of fiber function^2^,^23^,^24^,^25^,^26^. Answering these important questions in metabolic disease models can further lead to identification of bacterial and molecular markers associated with functional fiber.

In the current study, we employed a resistant maltodextrin (RM) to address the important questions whether gut microbiota are responsible for conferring the health benefits of RM and what microbial and metabolic signatures are associated with RM-mediated improvement in mouse metabolic disease models. Combining physiological, 16S sequencing and metabolomic approaches, our work demonstrates that the beneficial effects of RM are mediated through the metabolic function of gut microbiota, and reveals important microbial and metabolic markers for RM in metabolic disease.

## Results

### Resistant maltodextrin (RM) improves glycemic control

**Figure 1:**
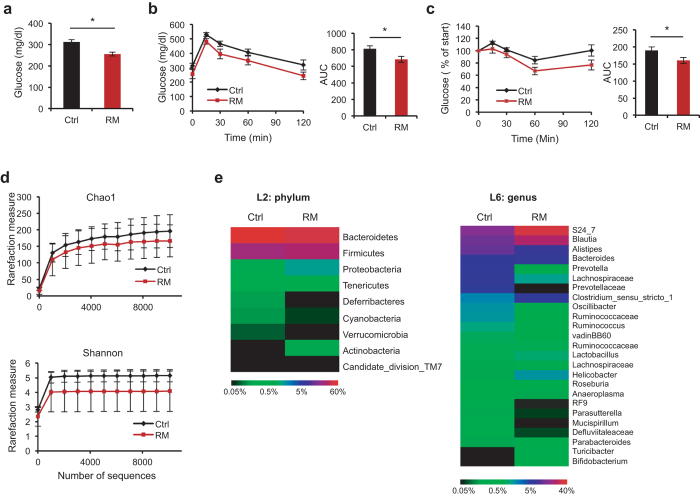
RM improves energy homeostasis and alters gut microbiota in *db/db* mice. Fasting glucose (**a**), oral glucose tolerance test (OGTT) (**b**) and insulin tolerance test (ITT) (**c**) showed improvements in *db/db* mice after 8 weeks of RM treatment relative to Ctrl (n = 7-8). Area under curve (AUC) values are also shown for GTT and ITT. Values are presented as means ± SEM. *p < 0.05, **p < 0.01. For microbial sequencing analysis, alpha-diversity plots of gut microbiota (**d**) and heat maps of relative abundance of OTUs (as percentage of total microbiota) (**e**) are shown for *db/db* mice treated for 8 weeks with Ctrl or RM (n = 4-5). Level 2 (phylum) and 6 (genus) are shown. P values for diversity plots were calculated by 2-way ANOVA (repeated measure) to be Chao1 (P = 0.62) or Shannon (P = 0.59). See Supplementary Tables 1 and 2 for numerical values for heat maps in (**e**).

To address the question whether resistant maltodextrin (RM) can improve metabolism in metabolic disease models, we employed a previously described soluble RM (Fibersol®-2) manufactured through a proprietary method involving controlled enzymatic treatment of corn starch^5^,^27^. Specifically, *db/db* mice (Jackson Laboratory), a commonly used type 2 diabetes model lacking a functional leptin receptor and consequently developing severe obesity and glucose intolerance, were fed with regular chow diet (Purina 5001) and either regular (Ctrl) or 1% (w/v) RM drinking water. Mice treated with RM over an 8-week period showed a modest trend toward a blunted weight gain and food intake during in RM-fed mice (Supplementary Fig. 1). Importantly, RM treatment led to significantly decreased fasting glucose levels (Fig. 1a). Furthermore, oral glucose tolerance test (OGTT) showed an improved glucose control in the RM group over Ctrl (Fig. 1b). Indicative of a sensitized insulin response, insulin tolerance test (ITT) also revealed improved glucose clearance in response to insulin injection in the RM group (Fig. 1c). To further substantiate the efficacy of RM in glycemic control, we utilized diet-induced obesity (DIO) mice as a second metabolic disease model. Wild-type C57B/6J mice (Jackson Laboratory) were treated with high-fat diet, in conjunction with regular drinking water (Ctrl) or 1% RM water. Consistent with results from *db/db* mice, we again observed reduced weight gain over the 8-week treatment period (Supplementary Fig. 2a). Likewise, glucose control was also improved in RM-treated DIO mice (Supplementary Fig. 2, b and c). Together, in accordance with previous findings^4^,^28^, our results from two complementary mouse metabolic disease models showed that RM exerts a significant role in glucose control.

### Gut microbial remodeling by RM

Dietary fibers are generally known as prebiotics, promoting gut content of probiotics such as *Lactobacillus* and *Bifidobacterium*. Previous studies have also shown remodeling of gut microbiota by RM, and RM can be fermented by gut microbiota for energy and signaling functions^5^. To begin to investigate whether RM improves glycemic control owing to gut microbial remodeling, we conducted Illumina-based 16S rRNA sequencing of fecal DNAs from Ctrl and RM treated *db/db* mice shown in Fig. 1. RM treatment did not significantly alter diversity^29^,^30^ (Fig. 1d) or phylum abundance (Fig. 1e, left panel) of gut microbiota in these severely obese and diabetic mice. However, a slight increase in the *Firmicutes/Bacteroidetes* ratio by RM treatment (Supplementary Table 1) correlates with improved glycemic control, consistent with previous human diabetes studies^31^ and distinct dietary association of these phyla^32^ (see also below).

In comparison, a number of notable changes were observed at the genus level (Fig. 1e, right). For example, a prebiotic role of RM was substantiated as both *Lactobacillus* and *Bifidobacterium* were found to be moderately elevated in abundance (Fig. 1e, right panel; Supplementary Table 2). Importantly, *Alistipes*, previously shown to be closely correlated with an animal fat/protein diet^32^, was markedly reduced in the RM group (8.86% vs. 16.10% in Ctrl; Supplementary Table 2). Similarly, the abundance of *Bacteroides*, a predominant genus in *Bacteroidetes*, was also diminished by RM, albeit to a lesser degree (20% reduction). These bacteria are bile compatible^32^, thus under negative selection pressure in fiber-enriched diets. Together, decreases in *Alistipes* and *Bacteroides* and concomitant increases in *Lactobacillus* and *Bifidobacterium* indicated that RM is a functional fiber with broad microbial remodeling activities.

### A causal role of gut microbiota in RM efficacy in glucose control

**Figure 2:**
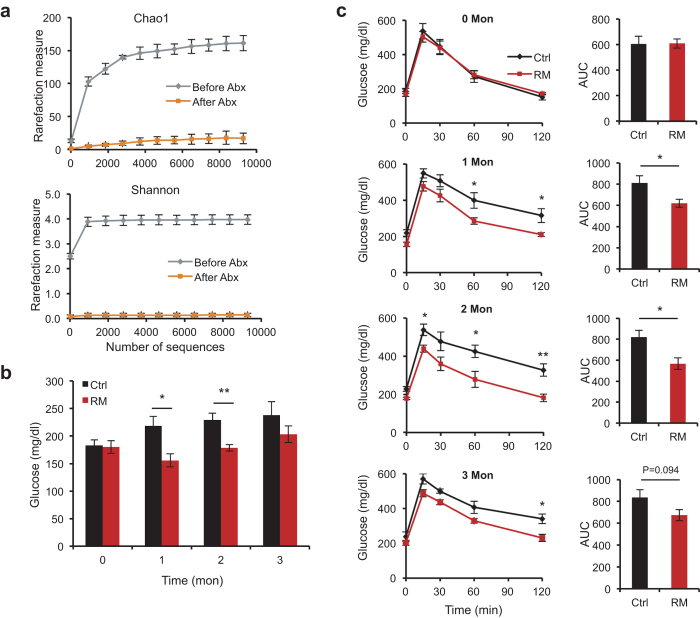
Fecal microbiota from donor *db/db* mice confers metabolic benefits of RM in recipient *db/db* mice. (**a**) Alpha-diversity plots showed depletion of gut microbiota by antibiotics treatment in recipient mice prior to fecal microbiota transplantation. P values were calculated by 2-way ANOVA (repeat measure) to be Chao1 (P < 0.0001) or Shannon (P < 0.0001). Fasting glucose (**b**) and oral glucose tolerance test (OGTT) (**c**) in recipient *db/db* mice before (0 month) and after transplantation (1, 2 and 3 months) of fecal microbiota from donor mice (n = 6). Area under curve (AUC) values are also shown. Values are presented as means ± SEM. *p < 0.05, **p < 0.01.

To directly address a causal role of gut microbial remodeling in mediating the beneficial effects of RM in glucose control, we conducted fecal microbiota transplantation (FMT) experiments^33^,^34^. Recipient *db/db* mice were first treated with an antibiotics cocktail to remove a vast majority of intrinsic gut bacteria (Fig. 2a, Supplementary Fig. 3 and Tables 3 & 4). Subsequently, fecal suspensions from either Ctrl or RM donor mice (Fig. 1) were administered to respective recipient mice, and glucose homeostasis was monitored throughout the experimental period. No significant effects on body weight were found (Supplementary Fig. 4a). In contrast, recipient mice that received fecal materials from RM donors showed a robust and progressive improvement in glucose tolerance up to 2 months post-FMT (Fig. 2, b & c). Interestingly, the peak glucose tolerance in recipient mice appeared to be more pronounced than that exhibited by donor mice (Fig. 1). By the end of 3 months post-FMT, however, the effects have subsided. We found improved insulin tolerance also at 2 months post-FMT (Supplementary Fig. 4b), coincident with the peak OGTT improvement. Of note, the recipient mice were never exposed to RM, indicating that gut microbiota in RM donors were sufficient to confer the efficacy of RM in glucose homeostasis in recipient mice.

### Transmissible microbial remodeling by RM

**Figure 3:**
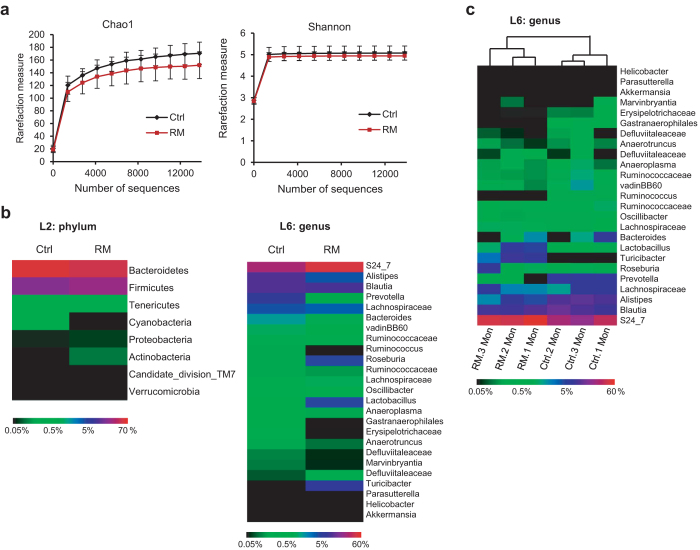
Transmissible remodeling of gut microbiota in recipient *db/db* mice after fecal microbiota transplantation. Alpha-diversity plots (**a**), heat maps of average (**b**) and time-resolved (**c**) abundance of phylum- and genus-level OTUs calculated as percentage of total microbiota in recipient *db/db* mice (n = 6) are shown. Values are presented as means ± SEM. P values for diversity plots were calculated by 2-way ANOVA (repeated measure) to be Chao1 (P = 0.014) or Shannon (P = 0.086). See Supplementary Tables 5 and 6 for numerical values for heat maps.

To gain insight into the significant improvement in glycemic control following FMT, we conducted 16S rRNA sequencing using fecal DNAs from recipient mice. Consistent with donors, RM recipients showed moderate reduction in diversity (Fig. 3a), and similar slight shifts in the abundance of *Firmicutes*, *Bacteroidetes* and *Actinobacteria* (Fig. 3b, left panel; Supplementary Table 5). Transmissible microbial remodeling can be more clearly characterized at the genus level (Fig. 3b, right panel; Supplementary Table 6). Specifically, among the 22 altered OTUs at the genus level, 14 were found to retain similar up- or down-regulation responses to RM as in donors, most notably *Alistipes*, *Bacteroides*, and *Lactobacillus* (Supplementary Tables 2 & 6). Among them, 12 showed changes that were significant or approaching significant (p values between 0.05 and 0.1) in both donors and recipients, whereas the other 2 were significant in either donors or recipients (Supplementary Table 6).

We further analyzed the dynamic microbial remodeling in recipient mice during the 3-month post-FMT period (Fig. 3c, Supplementary Fig. 5 and Tables 7 & 8). Interestingly, hierarchical clustering showed that the overall microbial patterns of RM recipients segregated from those of control samples, and that 1 and 2 months post-FMT were more similar than 3 months post-FMT (Fig. 3c). This pattern mirrors that of glucose tolerance as shown in Fig. 2c. Furthermore, quantitative comparison between RM and Ctrl samples in a time-resolved manner uncovered several genera, including *Lactobacillus* and *Bacteroides*, showing pronounced changes in parallel with the relative improvement in glucose tolerance over the 3-month period (Supplementary Table 8). Together, our analyses identified RM-associated persistent and transmissible microbial changes at both phylum and genus levels.

### Pronounced metabolomic shift in response to RM

**Figure 4:**
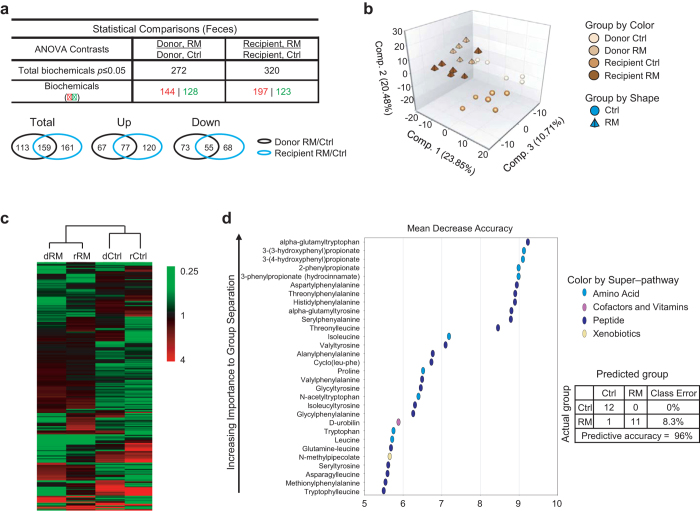
RM alters fecal metabolomic profiles in donor and recipient *db/db* mice. (**a**) Numbers of fecal metabolites affected, either up- or down-regulated (p < 0.05), by RM in donor and recipient mice (n = 6). Venn diagrams showing the overlaps between donor and recipient samples are also shown. (**b**) Principal component analysis of fecal metabolites affected by RM in donor and recipient mice (n = 6). (**c**) Hierarchical clustering heat map showing a predominant effect of RM on the relative abundance of the fecal metabolites such that RM donor and RM recipient mice (dRM and rRM), as well as Ctrl donor and Ctrl recipient (dCtrl and rCtrl), are clustered together (n = 6). Color bar values correspond to relative abundance measured in metabolomic analysis. Note that the color scheme is different from that for the heat maps showing microbial changes. (**d**) Random forest analysis showing a unique metabolomic signature between Ctrl and RM fecal samples, preserved in both donors and recipients, with a predictive accuracy of 96% in differentiating between the Ctrl and RM groups (n = 6).

To extend the above microbiome analysis and obtain functional insight into the glycemic effects of RM, we conducted global metabolomic profiling, using fecal samples from both donors and recipients at 2 months post-FMT, corresponding to peak metabolic efficacy (Fig. 2). A total of 727 compounds of known identity (named biochemicals) were identified (Supplementary Table 9), and large numbers (272 and 320 for donors and recipients respectively) of metabolites were found to be significantly altered in abundance as a result of RM treatment (Fig. 4a, upper panel). Venn diagrams further demonstrated significant overlaps of such changed metabolites between donors and recipients (Fig. 4a, lower panel). In accordance, principal component analysis (PCA; Fig. 4b) and hierarchical clustering (Fig. 4c) together illustrated both similarity and variance between donor vs. recipient samples with the same treatment. For example, whereas PC1 and PC2, explaining 23.85% and 20.48% of differences respectively, mainly distinguished Ctrl vs. RM treatment, PC3 (10.71%) appeared to correlate with donor vs. recipient identity (Fig. 4b).

Next, random forest analysis was conducted to pinpoint the group of metabolites most associated with RM and also preserved in both donors and recipients (Fig. 4d). Interestingly, 4 out of the 5 top metabolites identified were phenylpropionates and hydroxyphenylpropionates, products from catabolism of the aromatic amino acids phenylalanine and tyrosine with various physiological effects^2^,^35^,^36^. In accordance, phenylalanine and tyrosine metabolism was among the highly ranked metabolic pathways in both donor and recipient RM mice (Supplementary Fig. 6). However, note that phenylpropionates (enriched) and hydroxyphenylpropionates (depleted) were differentially affected in RM samples relative to Ctrl (Supplementary Table 10). Furthermore, levels of several other bacterial metabolites from aromatic amino acid breakdown such as p-cresol sulfate, phenol sulfate and 3-indoxyl sulfate were also reduced in RM donors and/or recipients (Supplementary Fig. 7). This latter group of phenolic and indoxyl acids have been linked with increased risk of cardiovascular disease, inflammation and oxidative damage^2^,^18^. Our results thus indicated a role of RM in disease prevention, and also suggested other sources than protein catabolism for the enrichment of phenylpropionates.

**Figure 5:**
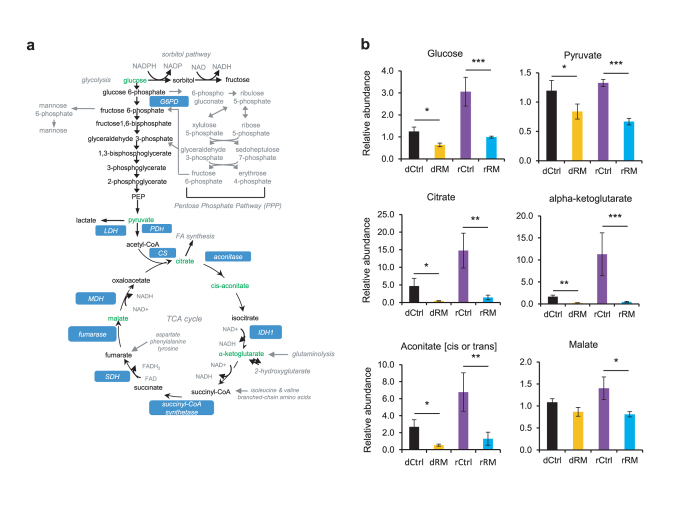
RM alters metabolites involving in glucose metabolism of gut microbiota in donor and recipient mice. (**a**) Schematic of the glucose metabolism pathway. Metabolites decreased by RM treatment in the feces of donor or recipient *db/db* mice are highlighted in green whereas metabolites not detected or unchanged are marked in black or grey. (**b**) Graphs showing relative abundance of metabolites affected by RM treatment in the feces of donor or recipient *db/db* mice (n = 6). Values are presented as means ± SEM.

In light of the improved energy metabolism in RM mice, we next examined metabolites related to sugar and lipid metabolism. Strikingly, glucose levels were found to be markedly attenuated in both RM donors and recipients compared with controls (Fig. 5). Furthermore, several intermediate metabolites for glucose metabolism, including pyruvate (glycolysis), citrate, α-ketoglutarate, aconitate and malate (TCA cycles) were also significantly reduced, strongly suggesting diminished glucose flux and improved energy homeostasis in the diabetic *db/db* mice.

**Figure 6:**
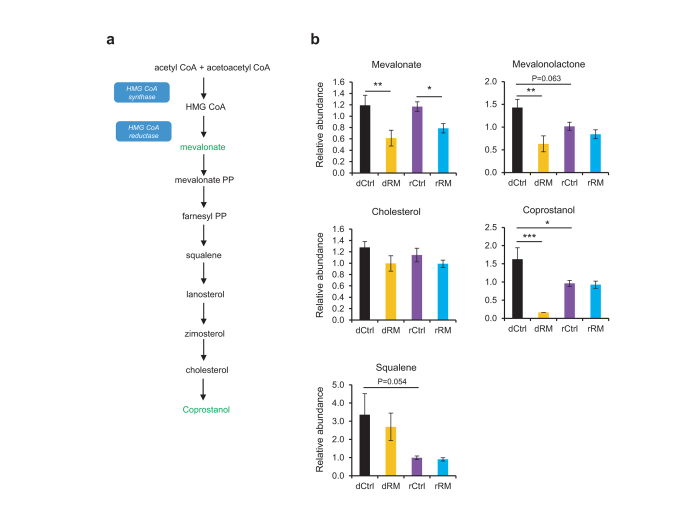
RM alters metabolites involving in cholesterol metabolism of gut microbiota in donor and recipient mice. (**a**) Schematic of the cholesterol metabolic pathway. Metabolites decreased by RM treatment in the feces of donor or recipient *db/db* mice are highlighted in green whereas metabolites not detected or unchanged are marked in black. (**b**) Graphs showing relative abundance of metabolites affected by RM treatment in the feces of donor or recipient *db/db* mice (n = 6). Values are presented as means ± SEM.

In addition to glucose metabolism, cholesterol metabolism was also found to be considerably improved by RM (Fig. 6). Levels of two intermediate metabolites, mevolonate and mevalonolactone, were significantly decreased in RM donor and recipient mice. In accordance with their decreases, the bacterial metabolic derivative coprostanol was also reduced in RM donors. Consistent with previous studies^6^, these results strongly indicated a beneficial role of RM in cholesterol control. Interestingly, coprostanol, squalene, and mevalonolactone showed diminished levels in rCtrl relative to dCtrl, perhaps reflecting beneficial effects of antibiotics treatment on controlling cholesterol biosynthesis^37^.

Together, the above metabolomic observations revealed profound metabolic benefits of RM or RM-derived microbiota.

## Discussion

Average fiber intake among Americans reaches only half of the recommended amounts^1^. Therefore, detailed functional and causal relationship studies will provide concrete scientific basis to raise fiber awareness and consumption. Resistant maltodextrin (RM) described herein is a soluble, non-viscous and fermentable dietary fiber, with minimal GI disturbance side effects^5^. In studies involving healthy human subjects, RM has been shown to improve colonic motility, fecal characteristics and probiotic (*Bifidobacterium*) population^5^.

In the current study, we investigated the metabolic efficacy of RM using both diet-induced obesity (DIO) and genetic diabetic *db/db* mice. Whereas its effects on body weight were moderate, with slightly more pronounced efficacy in DIO mice, we observed significant ameliorative effects of RM on glucose homeostasis in these mouse models. It is worth noting that such effects require prolonged treatment, 8 weeks in our study. In accordance with an improved glucose control as revealed by these physiological assays, metabolomic profiling showed much reduced levels in both glucose and several intermediate metabolites from glycolysis and the TCA cycle in RM donor and recipient mice. Furthermore, levels of cholesterol and several metabolites for cholesterol metabolism were also strongly reduced by RM or RM-derived microbiota, consistent with a reported hypocholesterolemic effect^6^,^38^. These complementary physiological and metabolomic studies provide strong evidence for a metabolic function of RM in pathophysiological settings, and highlight the importance of sustained exposure of fiber or fiber-derived microbiota.

Fecal microbiota transplantation (FMT) has been a highly effective clinical treatment for bowl diseases and more recently a powerful method to investigate a functional relationship between gut microbiota and physiological changes^17^,^39^. Adapting the procedure to diabetic *db/db* mice, we showed that antibiotics-treated recipient mice displayed profound improvement in glucose tolerance following FMT in a time-dependent manner, peaking at 2 months post-FMT. In conjunction with antibiotics treatment, the transplanted gut microbiota were fully capable of mediating RM metabolic efficiency, strongly suggesting a causal relationship between transmissible microbial remodeling and glycemic control. The subsequent attenuation of glucose tolerance at 3 months post-FMT suggests dynamic microbial changes associated with antibiotics and/or transplantation procedures^40^, underscoring the aforementioned requirement for sustained exposure to fiber or fiber-derived microbiota.

Dietary fibers as prebiotics have been well-documented^12^. In particular, RM has previously been shown to be moderately bifidogenic in healthy subjects^5^. Likewise, we observed increased levels of *Lactobacillus* and *Bifidobacterium* in RM donor and/or recipients, further substantiating a prebiotic function of RM. Besides probiotics, we also identified numerous other changes in microbial landscape as a result of RM. At the phylum level, RM led to a slight shift toward *Firmicutes* and *Actinobacteria* at the expense of *Bacteriodetes*. More significant changes at the genus level were determined. Notably, *Alistipes* was found to be markedly repressed. In a previous study comparing effects of animal vs plant derived diets on microbiota, *Alistipes* was shown to be enriched in the former, protein-rich diet, consistent with a purported role in protein fermentation. The depletion of *Alistipes* by RM in the current study is thus in accordance with the observed effects of plant-derived fiber-rich diet^32^, suggesting a beneficial role of RM to suppress putrefactive protein breakdown. While consistent with previous studies showing correlation between improved glycemic control and abundance shift of these major phyla^31^,^41^, these results are on the other hand at odds with several other observations^42^,^43^,^44^,^45^,^46^, suggesting a highly context-dependent and holistic nature of microbial remodeling in association with physiological changes. Functional assessment, such as metabolic flux, is needed for physiological interpretation of microbial remodeling^47^,^48^,^49^.

Our global metabolomic profiling revealed extensive metabolic flux, as indicated by the large number of metabolites (~300) showing RM-induced changes in abundance. Among the top-ranked metabolites associated with RM were phenylpropionates and hydroxyphenylpropionates. It is unlikely that the striking enrichment of phenylpropionates (>50 fold, RM/Ctrl) was a result of protein breakdown as levels of hydroxyphenylpropionates and several other amino acid metabolites (see below) were found to be strongly reduced. Two other metabolic pathways may contribute to phenylpropionate enrichment. First, dietary polyphenols are known to be catabolized to generate phenylpropionates^2^,^50^. The corn and oats in mouse chow diet contain rich polyphenols, and RM may enhance microbial degradation of the dietary polyphenols. Alternatively, propionate may also promote phenylpropionate synthesis. Propionate can be converted to phosphoenolpyruvate, which in turn serves as substrate for the Shikimate pathway in commensal bacteria to produce phenylalanine and thus ultimately phenylpropionates^51^,^52^,^53^. The untargeted metabolomic platform used in this study was not able to identify SCFAs due to their polarity and volatility. However, previous studies have demonstrated robust propionate/SCFA production from this RM^5^,^54^. Interestingly, propionate was recently shown to link central energy regulation with intestinal gluconeogenesis (IGN) to regulate energy metabolism^8^. Future studies will be important to further investigate these possibilities.

Beyond SCFAs, there are evidently extensive host-gut metabolic interactions^26^,^55^. To address the important questions regarding other metabolic changes associated with dietary fibers and their functional roles, we showed here that levels of several amino acid fermentation products including phenolic and indoxyl acids were significantly diminished in RM donor and recipient mice. This pattern is consistent with the depletion of *Alistipes*, previously shown to be associated with a high fat/protein diet^32^. These gut metabolic products are known to associate with higher disease risks^18^; thus, reduced levels of these bacterial byproducts suggest important health benefits of this fiber.

Combining physiological assays and metagenomic/metabolomic profiling, the current study reveals a key role of RM in glucose and cholesterol homeostasis and highlights the underlying profound microbial and metabolomic remodeling. Our study uncovers important changes in the abundance of microbial OTUs (e.g., probiotics and *Alistipes*) and fecal metabolites (phenylpropionates and glycolysis-TCA/cholesterol/amino acid intermediates). Future studies will focus on their functional mechanisms in RM-mediated metabolic regulation, which may ultimately lead to improved understanding and application of functional dietary fibers.

## Materials and Methods

### Mice and resistant maltodextrin

Animal husbandry for all the studies was carried out under IACUC guidelines and the procedures were conducted as described in an animal protocol approved by the University of Texas Health Science Center at Houston (UTHSC-H) and the University of Wisconsin at Parkside. Wild-type (WT) and *db/db* mice, on the C57BL/6J genetic background, were obtained from the Jackson Laboratory (#664 and #697, respectively). Verification genotyping was carried out according to Jackson Laboratory protocols by using 2x PCR master mix (GenDEPOT). Mice were routinely group-housed (2/cage for *db/db* mice and 2-4/cage for WT mice) in standard animal facility under a 12h/12h cycles. The resistant maltodextrin (Fibersol®-2), manufactured by Matsutani and ADM, has been previously described^5^,^27^.

### Mouse treatment and body weight measurements

Six-week-old *db/db* mice, fed with regular chow diet (Purina 5001), were randomly grouped to receive regular drinking water (Ctrl) or 1% RM *ad libitum* during the experimental period. Body weight was measured weekly. For diet-induced obesity, WT mice at 6 weeks of age were fed with HFD (Research Diets D12492) until the end of the experimental protocol. Mice were randomly divided into the control (Ctrl) group fed with regular drinking water and the RM group fed with 1% RM drinking water *ad libitum* during the experimental period.

### Food and drinking water intake

Food intake was determined by calculating the difference in food weight during 24 hr intervals. Three independent experiments were carried out to calculate the average food intake. For drinking water measurement, the volume of drinking water was measured once every two weeks. The drinking water intake was calculated from averaged volumes of drinking from four independent experiments.

### Oral glucose tolerance tests

Overnight fasted *db/db* and DIO mice were oral gavaged with 1 g/kg glucose, and glucose were measured from tail blood before and 15, 30, 60, or 120 min by using the ONETOUCH UltraMini blood glucose monitoring system (LifeScan).

### Insulin tolerance tests

Following 5 hr fasting, mice were injected intraperitoneally with 1.0 U/kg insulin (Sigma), and glucose levels were measured from tail blood before at 15, 30, 60, or 120 min by using the ONETOUCH UltraMini blood glucose monitoring system (LifeScan).

### Antibiotics treatment and fecal microbiota transplantation

Six-week-old *db/db* mice were treated with a cocktail of broad spectrum antibiotics (1 g/L ampicillin, neomycin, and metronidazole and 0.5 g/L vancomycin) in drinking water for 3-4 weeks^33^,^34^. The mice were allowed 3-4 days to recover before fecal microbiota transplantation started. Fresh fecal pellets were collected from donor *db/db* mice after two hours in collection cages with paper liner. Subsequently, 200 mg of pellets were weighed and resuspended and homogenzied at 1:10 (w/v) in transfer buffer (0.1 M phosphate buffered saline, pH 7.0, pre-reduced with 0.05% cysteine HCL. To each recipient mice, 100 μl of homogenates were used for oral gavage. The transplantation procedure was carried out every three days, four times total for each experiment. Throughout the entire experimental period, the mice were maintained on the regular chow diet (Purina 5001).

### 16S rRNA gene sequencing

Bacterial 16S rRNA gene profiling was conducted by the Alkek Center for Metagenomics and Microbiome Research at Baylor College of Medicine using Illumina MiSeq platform as previously described^56^. Briefly, fresh fecal pellets were collected from each cage on 15-20 min intervals and immediately frozen on dry ice. Pooled aliquots were stored at −80 °C at the end of 2 hr collection periods. Microbial DNA was extracted with PowerSoil® DNA Isolation kit (MoBio) following the manufacturer’s guidelines. The 16S rDNA V4 region amplicons (single index) were produced by PCR and sequenced on the MiSeq platform (Illumina) using the 2 × 250 bp protocol yielding pair-end reads that overlap by ~247 bps. Following sequencing, raw BCL files were retrieved from the MiSeq platform and called into fastqs by Casava v1.8.3 (Illumina). The read pairs were demultiplexed based on unique molecular barcodes, filtered for PhiX using Bowtie2, and reconstituted into two fastq files for each read using standard BASH. A barcodes file was generated from a raw fastq base called previously to preserve the original barcode qualities associated per read cluster. Sequencing reads were merged (allowing 4 mismatches per ≥50 bases) and processed using USEARCH v7.0.1001 (maximum error method)^57^. Sequences were demultiplexed using QIIME v1.8.0 and then clustered using the UPARSE pipeline^57^. Operational taxonomic unit (OTU) classification was achieved by mapping the UPARSE OTU table to the SILVA database. Abundances were recovered by mapping the demultiplexed reads to the UPARSE OTUs. A custom script constructed an OTU table from the output files generated in the previous two steps. The OTU table was used to calculate alpha-diversity, beta-diversity, provide taxonomic summaries, and in a variety of other analyses built into QIIME that allowed for the characterization of individual and group of samples based on alpha and beta diversity indices.

### Fecal metabolomic analysis

Global, untargeted metabolomic analysis was conducted by using Metabolon UPLC-MS/MS and GC-MS platform. Briefly, fresh fecal pellets were collected as above for 16S sequencing from either donor *db/db* mice after 2 months of RM treatment or recipient *db/db* mice 2 months after transplantation and stored at -80 °C. Frozen feces, six experimental samples from each group (dCtrl, dRM, rCtrl, and rRM), were lyophilized and weighed. Weight equivalents were then subjected to non-targeted metabolomic analysis platform including UPLC-MS/MS and GC/MS at Metabolon Inc.^58^. Identification and quantification of named metabolites were conducted based on previously published methods^59^. The metabolomic data were then analyzed by unsupervised principal component analysis to identify sets of patterns corresponding to uncorrelated variables. Such patterns, called principle components, can reveal metabolic distinction and similarity as a function of donor/recipient status and treatment. In the random forest analysis^60^ to identify biochemicals made the largest contribution to the classification of RM vs. Ctrl samples (donor and recipient combined), Mean Decrease Accuracy (MDA) was determined by randomly permuting a metabolite, running the observed values through a series of decision trees, and then reassessing the prediction accuracy. A predictive accuracy of 50% would be expected by chance, and a greater MDA score indicates stronger differentiating power.

### Statistical analyses

All data are presented as means ± SEM. Statistical significance was determined by one-way ANOVA (Dunnett’s test), two-way ANOVA repeated measures (Bonferroni’s test) and Mann-Whitney test. P < 0.05 was accepted as statistically significant. Statistical analyses were performed using the SigmaStat3.5 software (for ANOVA) and the Wilcox.test program of R (for Mann-Whitney). For metabolic studies, the N numbers refer to mouse numbers. For 16S sequencing and metabolomic profiling, the N numbers refer to independent fecal samples.

## Additional Information

**How to cite this article**: He, B. *et al.* Transmissible microbial and metabolomic remodeling by soluble dietary fiber improves metabolic homeostasis. *Sci. Rep.* **5**, 10604; doi: 10.1038/srep10604 (2015).

## Supplementary Material

Supplementary Information
