# Supplementary material for: Transmissible microbial and metabolomic remodeling by soluble dietary fiber improves metabolic homeostasis
Source: Sci Rep. 2015 Jun 4;5:10604. doi: 10.1038/srep10604 (PMC4455235; doi:10.1038/srep10604)
Supplement: Supplementary Information [file srep10604-s1.pdf]

Supplementary Information

Transmissible microbial and metabolomic remodeling by soluble dietary fiber improves metabolic homeostasis

Baokun He[1], Kazunari Nohara[1], Nadim J. Ajami[2], Ryan D. Michalek[3], Xiangjun Tian[2], Matthew Wong[2], Susan H. Losee-Olson[4], Joseph F. Petrosino[2], Seung-Hee Yoo[1], Kazuhiro Shimomura[5], Zheng Chen[1,*]

1. Department of Biochemistry and Molecular Biology, The University of Texas Health Science Center at Houston, 6431 Fannin St., Houston, TX 77030
2. The Alkek Center for Metagenomics and Microbiome Research, Department of Molecular Virology and Microbiology, One Baylor Plaza, Baylor College of Medicine, Houston, TX 77030
3. Metabolon Inc., 617 Davis Drive, Durham, NC 27713
4. RhythMed, 14673 W Birch Lane, Wadsworth, IL 60083
5. Matsutani America Inc., 500 Park Blvd. Suite 1240, Itasca, IL 60143

*: To whom correspondence should be addressed.

E-mail: Zheng.chen.1@uth.tmc.edu .

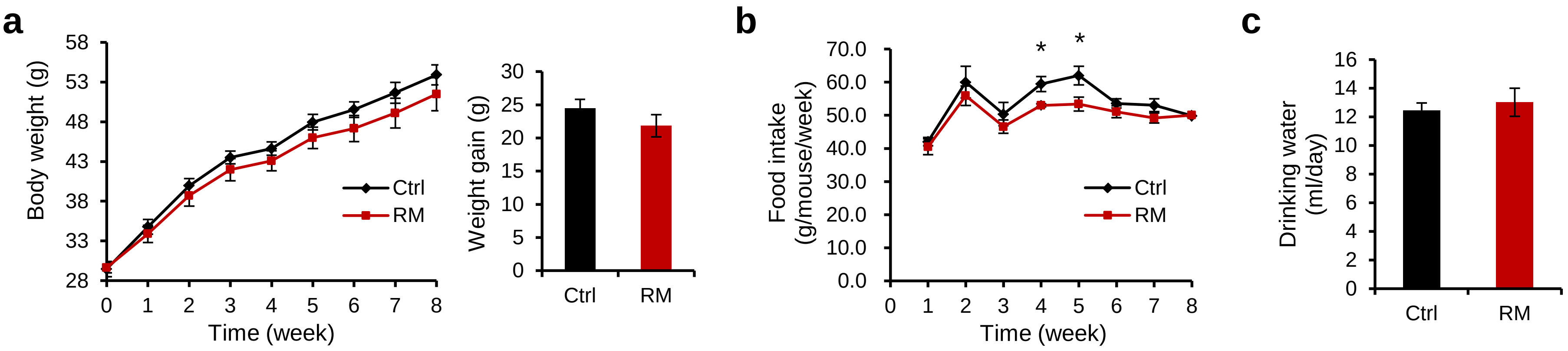

Supplementary Figure 1. Effects of RM on energy homeostasis in *db/db* mice. Body weight gain (**a**), food intake (**b**) and drinking water intake (**c**) in *db/db* mice after 8 weeks of Ctrl or RM treatment (n=4-8). Values are presented as means ± SEM. * $p < 0.05$.

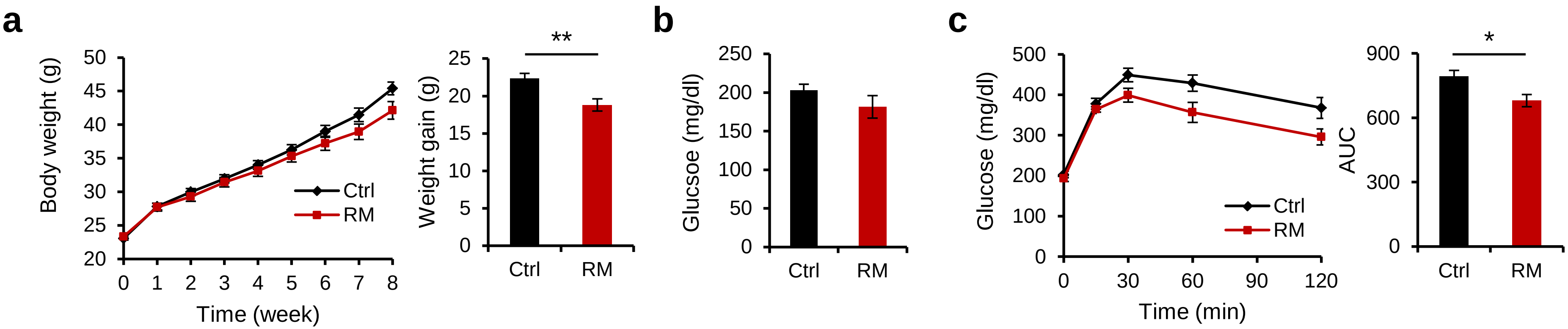

Supplementary Figure 2. Effects of RM on energy homeostasis in DIO mice. Body weight gain (**a**), fasting glucose (**b**), and oral glucose tolerance test (**c**) in DIO mice after 8 weeks of Ctrl or RM treatment (n=9-15). Values are presented as means ± SEM. * $p < 0.05$. ** $p < 0.01$.

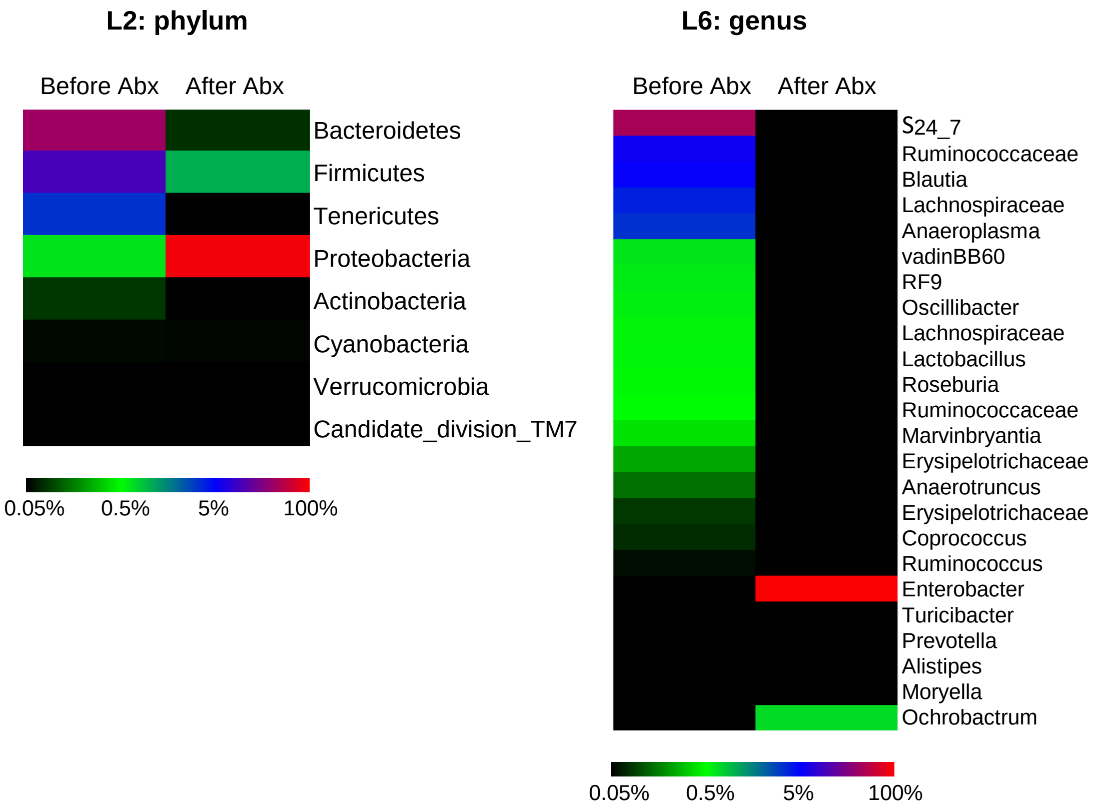

Supplementary Figure 3. Antibiotics treatment of recipient mice. Heat maps, showing the relative abundance of OTUs, indicate depletion of gut microbiota in antibiotics-treated *db/db* mice at both phylum (level 2) and genus (level 6) levels. See Supplementary Tables 3 and 4 for numerical values.

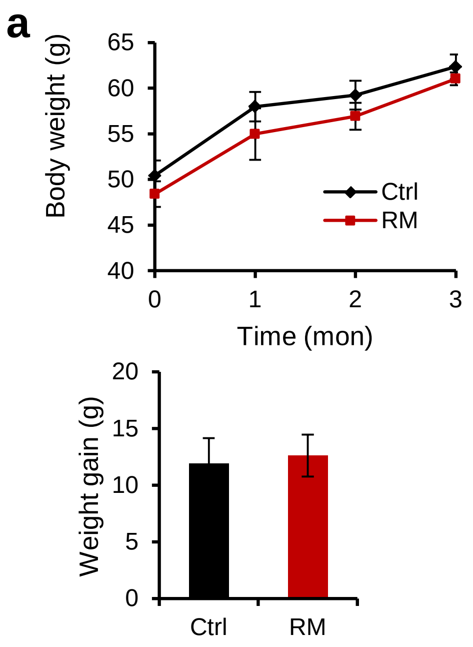

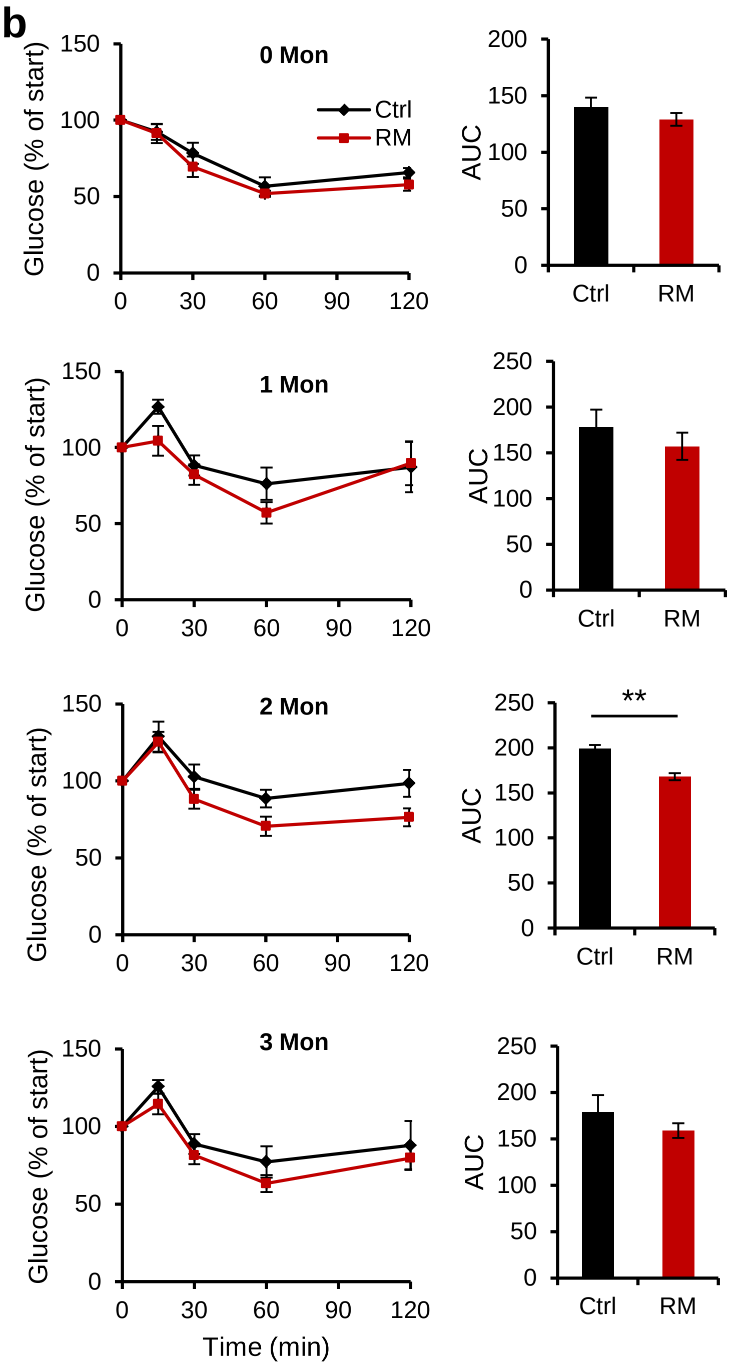

Supplementary Figure 4. Metabolic changes in recipient *db/db* mice after fecal microbiota transplantation (FMT). Body weight gain (**a**) and insulin tolerance test (**b**) in recipient *db/db* mice before transplantation (0 month) and after transplantation (1, 2 and 3 months) of gut microbiota (n=6). Values are presented as means ± SEM. ** $p < 0.01$.

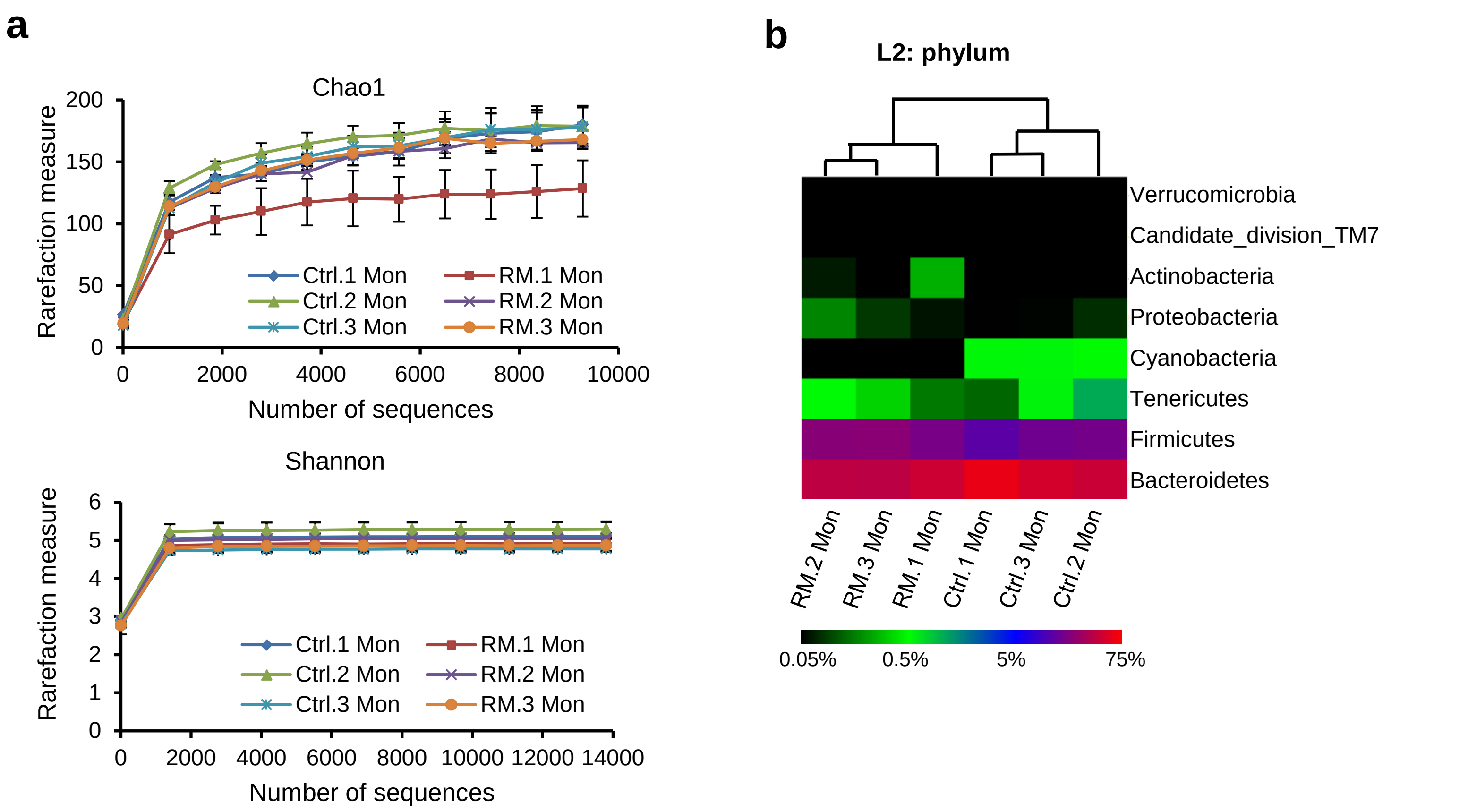

Supplementary Figure 5. Gut microbiota in recipient *db/db* mice after transplantation. Shannon alpha-diversity (**a**) and heat map of phylum-level gut microbiota changes (**b**) in recipient *db/db* mice 1, 2, and 3 months after transplantation (n=6). The heat map show the abundance of phylum-level OTUs calculated as percentage of total microbiota. Values are presented as means ± SEM. We performed 2-way ANOVA (repeated measure) analysis to compare diversity in Ctrl and RM samples. At 1, 2, and 3 months post-transplantation, Chao1: P=0.020, 0.063 and 0.45, respectively; Shannon: P=0.08, 0.30 and 0.31, respectively. See supplementary Tables 7 and 8 for numerical values for the heat maps.

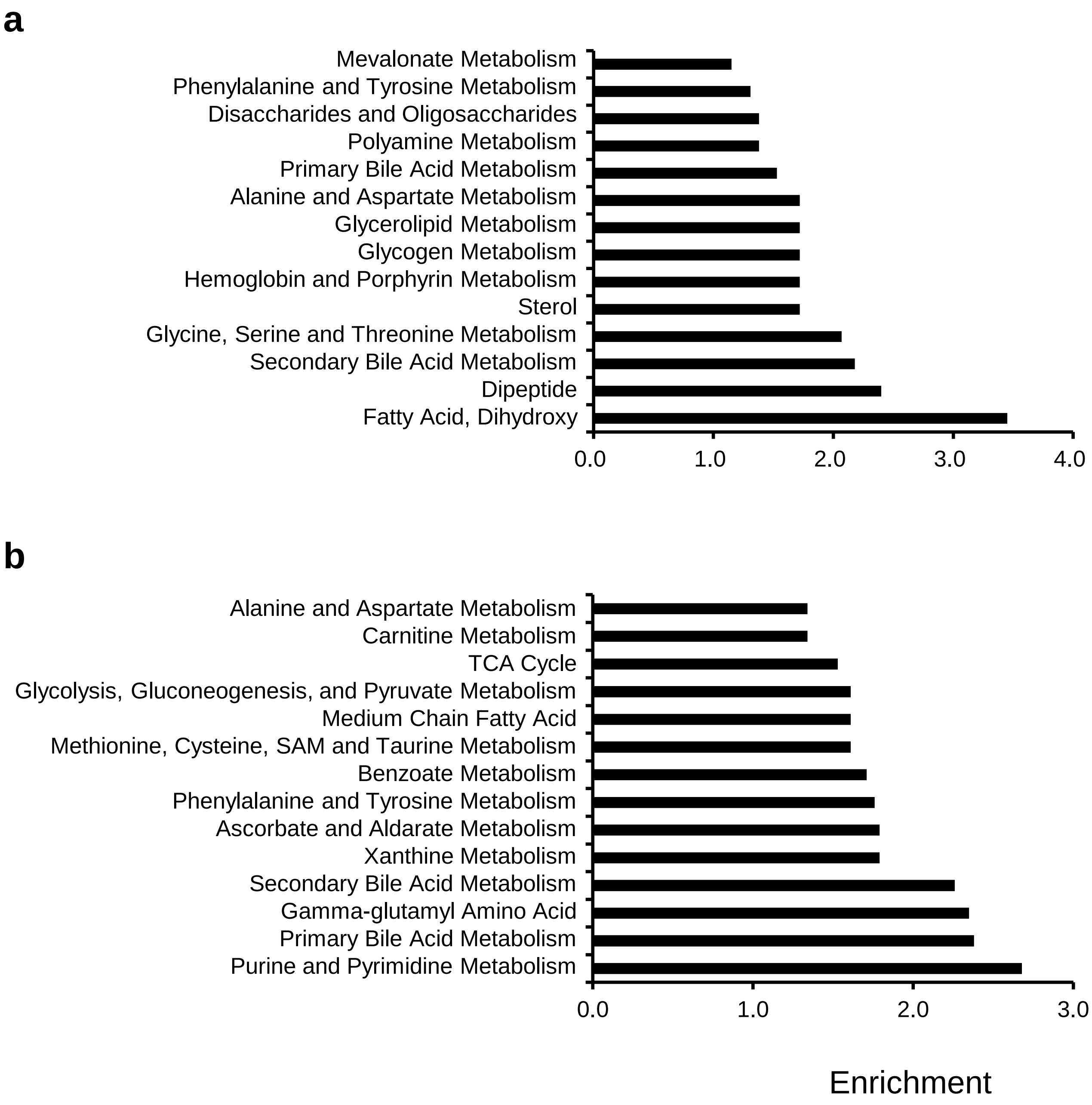

Supplementary Figure 6. Pathway Enrichment analysis revealed metabolic pathways affected by RM in donor and recipient *db/db* mice. A pathway enrichment value greater than one indicates that the pathway contains more experimentally regulated compounds relative to the study overall, suggesting that the pathway may be a target of interest of the experimental perturbation. Top 14 affected metabolic pathways are listed for donor (**a**) and recipient (**b**) *db/db* mice.

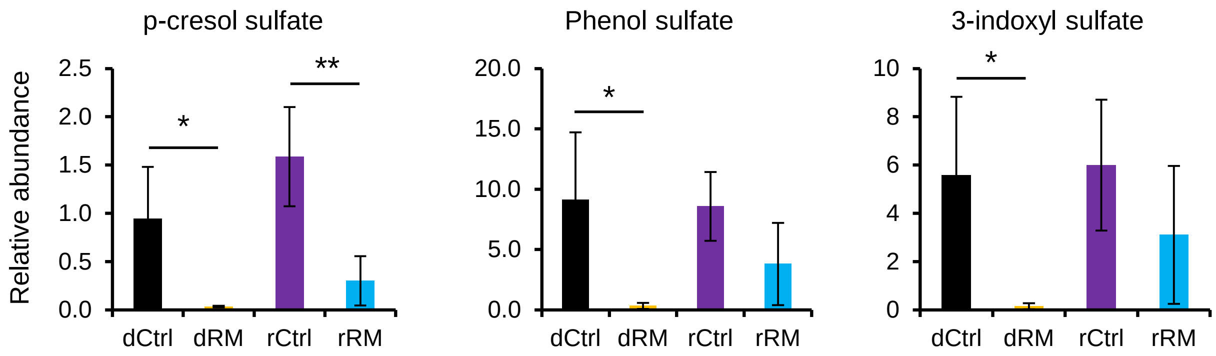

Supplementary Figure 7. RM amino acid gut-related metabolites of gut microbiota in donor and recipient mice. The signal intensity of metabolites affected by RM treatment in feces of donor or recipient *db/db* mice is shown (n=6). Values are presented as means ± SEM. * $p < 0.05$.

Supplemental Table 1. Average abundance of phylum-level OTUs in donor *db/db* mice treated with regular (Ctrl) or RM drinking water. The values were calculated as percentage of total microbiota in 4 pooled fecal samples collected from 8 group-housed donor mice for each treatment group. P values were calculated from 1-way ANOVA.

Taxon (Phylum level)	Ctrl (%)	RM (%)	Ctrl (SEM)	RM (SEM)	p-value
Bacteroidetes	55.894	52.179	1.457	6.173	0.286
Firmicutes	37.956	41.355	3.865	6.650	0.140
Proteobacteria	1.467	2.533	0.023	0.182	0.001
Tenericutes	1.102	0.464	0.380	0.214	0.026
Deferribacteres	0.276	0.000	0.276	0.000	0.092
Cyanobacteria	0.262	0.114	0.079	0.107	0.069
Verrucomicrobia	0.157	0.000	0.157	0.000	0.092
Actinobacteria	0.034	0.426	0.016	0.377	0.083
Candidate_division_TM7	0.027	0.008	0.017	0.004	0.070

Supplementary Table 2. Average abundance of genus-level OTUs in donor *db/db* mice treated with regular (Ctrl) or RM drinking water.The values were calculated as percentage of total microbiota from 4 pooled fecal samples collected from 8 group-housed donor mice for each treatment group. P values were calculated from 1-way ANOVA.

Taxon (Genus level)	Ctrl (%)	RM (%)	Ctrl (SEM)	RM (SEM)	p-value
S24_7	20.404	35.560	3.266	3.639	0.021
Blautia	17.261	28.363	1.310	0.672	0.001
Alistipes	16.104	8.860	3.271	2.211	0.010
Bacteroides	6.880	5.528	0.517	0.356	0.042
Prevotella	6.009	0.594	1.215	0.348	0.001
Lachnospiraceae	5.157	2.470	0.770	0.500	0.026
Prevotellaceae	4.847	0.000	4.197	0.000	0.060
Clostridium_sensu_stricto_1	3.091	7.225	3.091	5.843	0.258
Oscillibacter	2.788	0.370	1.076	0.031	0.035
Ruminococcaceae	2.743	0.751	1.176	0.279	0.016
Ruminococcus	2.270	0.507	0.701	0.232	0.003
vadinBB60	1.919	0.593	0.565	0.381	0.008
Ruminococcaceae	1.699	1.676	0.713	1.106	0.973
Lactobacillus	1.670	2.096	0.854	1.716	0.673
Lachnospiraceae	1.297	0.577	0.410	0.081	0.014
Helicobacter	1.254	2.803	1.086	2.428	0.228
Roseburia	0.964	0.938	0.065	0.472	0.959
Anaeroplasma	0.644	0.374	0.313	0.181	0.185
RF9	0.569	0.073	0.247	0.059	0.008
Parasutterella	0.382	0.112	0.331	0.097	0.464
Mucispirillum	0.367	0.000	0.318	0.000	0.060
Defluviitaleaceae	0.363	0.125	0.040	0.096	0.004
Parabacteroides	0.303	0.651	0.262	0.651	0.359
Turicibacter	0.033	0.557	0.019	0.557	0.109
Bifidobacterium	0.000	0.489	0.000	0.406	0.274

Supplementary Table 3. Average abundance of phylum-level OTUs in *db/db* mice treated with drinking water containing antibiotics. The values were calculated as percentage of total microbiota from 4-8 pooled fecal samples independently collected from 6 pair-housed recipient mice. P values were calculated by 1-way ANOVA.

Taxon (Phylum level)	Before Abx (%)	After Abx (%)	Before Abx (SEM)	After Abx (SEM)	p-value
Bacteroidetes	63.519	0.136	4.274	0.124	0.001
Firmicutes	31.140	1.923	3.895	1.878	0.001
Tenericutes	4.131	0.000	1.000	0.000	0.001
Proteobacteria	0.993	95.536	0.971	4.346	0.001
Actinobacteria	0.147	0.021	0.129	0.018	0.352
Cyanobacteria	0.066	0.059	0.061	0.031	0.914
Verrucomicrobia	0.003	0.002	0.002	0.001	0.715
Candidate_division_TM7	0.001	0.000	0.001	0.000	0.334

Supplementary Table 4. Average abundance of genus-level OTUs in *db/db* mice treated with drinking water containing antibiotics. The values were calculated as percentage of total microbiota from 4-8 pooled fecal samples independently collected from 6 pair-housed recipient mice. P values were calculated from 1-way ANOVA.

Taxon (Genus level)	Before Abx (%)	After Abx (%)	Before Abx (SEM)	After Abx (SEM)	p-value
S24_7	67.464	0.009	1.888	0.005	0.001
Ruminococcaceae	10.350	0.005	0.689	0.003	0.001
Blautia	6.520	0.005	0.824	0.002	0.001
Lachnospiraceae	4.449	0.003	0.858	0.002	0.001
Anaeroplasma	4.144	0.000	0.373	0.000	0.001
vadinBB60	0.969	0.000	0.072	0.000	0.001
RF9	0.855	0.000	0.269	0.000	0.007
Oscillibacter	0.769	0.000	0.196	0.000	0.002
Lachnospiraceae	0.660	0.001	0.111	0.001	0.001
Lactobacillus	0.658	0.002	0.444	0.001	0.162
Roseburia	0.620	0.000	0.226	0.000	0.016
Ruminococcaceae	0.546	0.000	0.075	0.000	0.001
Marvinbryantia	0.449	0.000	0.046	0.000	0.001
Erysipelotrichaceae	0.346	0.000	0.101	0.000	0.004
Anaerotruncus	0.251	0.000	0.037	0.000	0.001
Erysipelotrichaceae	0.150	0.000	0.034	0.000	0.001
Coprococcus	0.129	0.001	0.032	0.001	0.001
Ruminococcus	0.074	0.000	0.028	0.000	0.021
Enterobacter	0.003	98.530	0.001	0.433	0.001
Turicibacter	0.001	0.001	0.001	0.001	0.717
Prevotella	0.000	0.000	0.000	0.000	1.000
Alistipes	0.000	0.002	0.000	0.002	0.001
Moryella	0.000	0.000	0.000	0.000	1.000
Ochrobactrum	0.000	1.136	0.000	0.391	0.011

Supplementary Table 5. Average abundance of phylum-level OTUs in recipient *db/db* mice following transplantation of fecal microbiota from Ctrl or RM donor *db/db* mice. The values were calculated as percentage of total microbiota in 18 pooled fecal samples collected from 6 pair-housed recipient mice, with 6 samples each collected at 1-, 2-, and 3-month post-transplantation for each group. Sequencing results from these samples were combined to indicate overall recipient microbiota. P values were calculated from 1-way ANOVA.

Taxon (Phylum level)	Ctrl(%)	RM(%)	Ctrl(SEM)	RM(SEM)	p-value
Bacteroidetes	64.106	58.836	2.276	3.197	0.188
Firmicutes	34.179	40.485	2.158	3.131	0.100
Tenericutes	0.979	0.343	0.309	0.111	0.061
Cyanobacteria	0.614	0.010	0.119	0.002	0.001
Proteobacteria	0.080	0.116	0.017	0.039	0.402
Actinobacteria	0.024	0.199	0.007	0.081	0.040
Candidate_division_TM7	0.018	0.011	0.006	0.005	0.379
Verrucomicrobia	0.000	0.000	0.000	0.000	0.143

Supplementary Table 6. Average abundance of phylum-level OTUs in recipient *db/db* mice following transplantation of fecal microbiota from Ctrl or RM donor *db/db* mice. The values were calculated as percentage of total microbiota in 18 pooled fecal samples collected from 6 pair-housed recipient mice, with 6 samples each collected at 1-, 2-, and 3-month post-transplantation for each group. Sequencing results from these samples were combined to indicate overall recipient microbiota. P values were calculated from 1-way ANOVA. Transimissible changes, showing same patterns as in donors with p values at significant or approaching significant levels (between 0.05 and 0.1), are marked in red. Oscillibacter and Lactobacillus, showing the same trend in both donors and recipients but reached statistical significance in only one group, are marked in green.

Taxon (Genus level)	Ctrl (%)	RM (%)	Ctrl (SEM)	RM (SEM)	p-value
S24_7	39.558	53.174	2.477	3.212	0.020
Alistipes	18.135	4.236	1.265	0.744	0.001
Blautia	18.006	15.689	1.754	1.435	0.314
Prevotella	6.409	0.688	0.552	0.164	0.001
Lachnospiraceae	4.276	3.965	0.510	0.595	0.694
Bacteroides	2.607	1.671	0.587	0.349	0.075
vadinBB60	1.901	0.700	0.359	0.247	0.009
Ruminococcaceae	1.493	0.531	0.161	0.076	0.001
Ruminococcus	1.238	0.002	0.275	0.002	0.001
Roseburia	1.223	4.473	0.206	1.996	0.115
Ruminococcaceae	1.192	0.268	0.247	0.018	0.001
Lachnospiraceae	1.113	1.738	0.165	0.272	0.057
Oscillibacter	1.062	0.785	0.144	0.126	0.155
Lactobacillus	0.977	4.466	0.187	0.781	0.001
Anaeroplasma	0.921	0.375	0.315	0.110	0.112
Gastranaerophilales	0.610	0.000	0.119	0.000	0.001
Erysipelotrichaceae	0.356	0.053	0.068	0.015	0.001
Anaerotruncus	0.327	0.190	0.051	0.037	0.036
Defluviitaleaceae	0.223	0.091	0.046	0.020	0.012
Marvinbryantia	0.207	0.086	0.109	0.024	0.285
Defluviitaleaceae	0.149	0.357	0.044	0.081	0.030
Turicibacter	0.001	7.052	0.001	1.661	0.001
Parasutterella	0.000	0.000	0.000	0.000	1.000
Helicobacter	0.000	0.000	0.000	0.000	1.000
Akkermansia	0.000	0.000	0.000	0.000	1.000

Supplementary Table 7. Average time-resolved abundance of phylum-level OTUs in recipient *db/db* mice following transplantation of fecal microbiota from Ctrl or RM donor *db/db* mice. The values were calculated as percentage of total microbiota in 6 pooled fecal samples collected from 6 pair-housed recipient mice at each of the three time points (1-, 2-, and 3-month) post-transplantation for each group. P values were calculated from 1-way ANOVA.

Taxon (Phylum level)	Ctrl.1 Mon (%)	Ctrl.2 Mon (%)	Ctrl.3 Mon (%)	RM.1 Mon (%)	RM.2 Mon (%)	RM.3 Mon (%)
Bacteroidetes	69.167	60.189	62.962	61.224	56.633	56.447
Firmicutes	29.895	37.035	35.607	38.039	42.358	42.931
Cyanobacteria	0.627	0.570	0.645	0.015	0.004	0.004
Tenericutes	0.232	1.996	0.709	0.264	0.602	0.423
Proteobacteria	0.052	0.130	0.058	0.084	0.285	0.149
Actinobacteria	0.017	0.041	0.014	0.361	0.097	0.037
Candidate_division_TM7	0.009	0.040	0.005	0.013	0.020	0.008
Verrucomicrobia	0.000	0.000	0.000	0.000	0.000	0.001

Taxon (Phylum level)	Ctrl.1 Mon (SEM)	Ctrl.2 Mon (SEM)	Ctrl.3 Mon (SEM)	RM.1 Mon (SEM)	RM.2 Mon (SEM)	RM.3 Mon (SEM)
Bacteroidetes	4.547	2.163	4.186	4.111	6.726	6.740
Firmicutes	4.472	2.328	4.039	3.730	6.638	6.715
Cyanobacteria	0.094	0.191	0.330	0.002	0.003	0.003
Tenericutes	0.140	0.650	0.235	0.153	0.288	0.096
Proteobacteria	0.018	0.039	0.009	0.031	0.080	0.048
Actinobacteria	0.009	0.016	0.003	0.230	0.017	0.010
Candidate_division_TM7	0.002	0.014	0.004	0.008	0.013	0.007
Verrucomicrobia	0.000	0.000	0.000	0.000	0.000	0.001

Taxon (Phylum level)	RM.1 Mon/Ctrl.1 Mon (p value)	RM.2 Mon/Ctrl.2 Mon (p value)	RM.3 Mon/Ctrl.3 Mon (p value)
Bacteroidetes	0.224	0.626	0.431
Firmicutes	0.192	0.467	0.372
Cyanobacteria	0.001	0.014	0.080
Tenericutes	0.884	0.078	0.278
Proteobacteria	0.401	0.114	0.093
Actinobacteria	0.165	0.040	0.050
Candidate_division_TM7	0.617	0.320	0.724
Verrucomicrobia	1.000	1.000	0.341

Supplementary Table 8. Average time-resolved abundance of genus-level OTUs in recipient *db/db* mice following transplantation of fecal microbiota from Ctrl or RM donor *db/db* mice. The values were calculated as percentage of total microbiota in 6 pooled fecal samples collected from 6 pair-housed recipient mice at each of the three time points (1-, 2-, and 3-month) post-transplantation for each group. P values and difference in abundance between Ctrl and RM samples (RM-Ctrl) are calculated and shown below. Select bacteria showing time-dependent abundance changes are marked in red. P values were calculated from 1-way ANOVA.

Taxon (Genus level)	Ctrl.1 Mon (%)	Ctrl.2 Mon (%)	Ctrl.3 Mon (%)	RM.1 Mon (%)	RM.2 Mon (%)	RM.3 Mon (%)
S24_7	46.397	33.196	39.082	56.821	50.311	52.391
Alistipes	16.526	20.272	17.607	4.397	5.300	3.012
Blautia	13.397	17.390	23.230	18.559	13.539	14.971
Prevotella	6.241	6.716	6.270	0.000	1.022	1.042
Lachnospiraceae	5.058	5.233	2.539	3.075	3.121	5.699
Bacteroides	4.975	2.322	0.002	2.948	1.732	0.000
Ruminococcaceae	1.964	1.057	1.459	0.433	0.426	0.732
Ruminococcus	1.383	1.161	1.171	0.001	0.005	0.001
vadinBB60	1.329	2.685	1.690	0.232	1.329	0.538
Lactobacillus	1.287	0.974	0.668	4.561	6.811	2.028
Roseburia	1.214	1.669	0.787	1.770	0.609	11.041
Ruminococcaceae	1.101	2.160	0.314	0.249	0.270	0.286
Lachnospiraceae	0.691	1.383	1.266	1.629	1.757	1.827
Erysipelotrichaceae	0.634	0.214	0.220	0.068	0.070	0.021
Gastranaerophilales	0.627	0.559	0.644	0.000	0.000	0.000
Marvinbryantia	0.601	0.013	0.007	0.031	0.192	0.036
Oscillibacter	0.533	1.433	1.222	1.281	0.304	0.769
Anaerotruncus	0.220	0.515	0.247	0.102	0.184	0.283
Anaeroplasma	0.189	1.965	0.607	0.249	0.595	0.281
Defluviitaleaceae	0.036	0.348	0.285	0.031	0.086	0.157
Defluviitaleaceae	0.009	0.333	0.106	0.446	0.500	0.124
Turicibacter	0.001	0.000	0.001	5.169	12.457	3.529
Parasutterella	0.000	0.000	0.000	0.000	0.000	0.000
Helicobacter	0.000	0.000	0.000	0.000	0.000	0.000
Akkermansia	0.000	0.000	0.000	0.000	0.000	0.001

Taxon (Genus level)	Ctrl.1 Mon (SEM)	Ctrl.2 Mon (SEM)	Ctrl.3 Mon (SEM)	RM.1 Mon (SEM)	RM.2 Mon (SEM)	RM.3 Mon (SEM)
S24_7	3.294	2.974	4.318	4.108	7.194	6.125
Alistipes	1.146	3.320	1.674	0.162	2.116	0.833
Blautia	2.549	1.314	3.053	0.753	2.966	2.972
Prevotella	1.267	1.239	0.404	0.000	0.146	0.192
Lachnospiraceae	1.085	0.339	0.229	0.555	0.789	1.207
Bacteroides	0.351	0.101	0.001	0.203	0.052	0.000
Ruminococcaceae	0.320	0.198	0.087	0.119	0.044	0.162

Ruminococcus	0.307	0.848	0.089	0.001	0.005	0.001
vadinBB60	0.540	0.577	0.682	0.132	0.641	0.175
Lactobacillus	0.398	0.383	0.119	0.863	1.357	0.553
Roseburia	0.308	0.478	0.124	0.253	0.181	4.684
Ruminococcaceae	0.280	0.135	0.055	0.047	0.035	0.009
Lachnospiraceae	0.112	0.361	0.253	0.404	0.573	0.559
Erysipelotrichaceae	0.097	0.024	0.047	0.040	0.016	0.015
Gastranaerophilales	0.094	0.192	0.330	0.000	0.000	0.000
Marvinbryantia	0.229	0.003	0.003	0.018	0.008	0.012
Oscillibacter	0.110	0.155	0.207	0.080	0.027	0.090
Anaerotruncus	0.060	0.080	0.031	0.005	0.024	0.093
Anaeroplasma	0.139	0.670	0.229	0.144	0.289	0.072
Defluviitaleaceae	0.016	0.055	0.041	0.014	0.027	0.027
Defluviitaleaceae	0.006	0.047	0.026	0.121	0.168	0.032
Turicibacter	0.001	0.000	0.001	2.370	2.351	2.038
Parasutterella	0.000	0.000	0.000	0.000	0.000	0.000
Helicobacter	0.000	0.000	0.000	0.000	0.000	0.000
Akkermansia	0.000	0.000	0.000	0.000	0.000	0.001

Taxon (Genus level)	RM.1 Mon/Ctrl.1 Mon (p value)	RM.2 Mon/Ctrl.2 Mon (p value)	RM.3 Mon/Ctrl.3 Mon (p value)
S24_7	0.076	0.053	0.106
Alistipes	0.001	0.003	0.001
Blautia	0.081	0.263	0.081
Prevotella	0.001	0.001	0.001
Lachnospiraceae	0.135	0.034	0.028
Bacteroides	0.001	0.001	0.100
Ruminococcaceae	0.001	0.011	0.003
Ruminococcus	0.001	0.203	0.001
vadinBB60	0.076	0.147	0.133
Lactobacillus	0.006	0.002	0.037
Roseburia	0.194	0.065	0.054
Ruminococcaceae	0.013	0.001	0.625
Lachnospiraceae	0.049	0.593	0.382
Erysipelotrichaceae	0.001	0.001	0.002
Gastranaerophilales	0.001	0.015	0.080
Marvinbryantia	0.033	0.001	0.045
Oscillibacter	0.001	0.001	0.073
Anaerotruncus	0.078	0.003	0.718
Anaeroplasma	0.772	0.900	0.204
Defluviitaleaceae	0.800	0.002	0.027
Defluviitaleaceae	0.005	0.361	0.661
Turicibacter	0.054	0.001	0.114
Parasutterella	1.000	1.000	1.000

Helicobacter	1.000	1.000	1.000
Akkermansia	1.000	1.000	1.000

Taxon (Genus level)	RM-Ctrl (%) 1 Mon	RM-Ctrl (%) 2 Mon	RM-Ctrl (%) 3 Mon
S24_7	10.424	17.115	13.309
Alistipes	-12.129	-14.972	-14.595
Blautia	5.161	-3.851	-8.259
Prevotella	-6.241	-5.694	-5.228
Lachnospiraceae	-1.982	-2.111	3.160
Bacteroides	-2.027	-0.590	-0.002
Ruminococcaceae	-1.531	-0.631	-0.727
Ruminococcus	-1.382	-1.156	-1.170
vadinBB60	-1.098	-1.356	-1.152
Lactobacillus	3.273	5.836	1.360
Roseburia	0.556	-1.060	10.254
Ruminococcaceae	-0.852	-1.890	-0.028
Lachnospiraceae	0.938	0.374	0.561
Erysipelotrichaceae	-0.566	-0.144	-0.199
Gastranaerophilales	-0.627	-0.559	-0.644
Marvinbryantia	-0.570	0.178	0.029
Oscillibacter	0.748	-1.129	-0.453
Anaerotruncus	-0.117	-0.331	0.036
Anaeroplasma	0.060	-1.370	-0.326
Defluviitaleaceae	-0.006	-0.263	-0.127
Defluviitaleaceae	0.437	0.167	0.019
Turicibacter	5.168	12.457	3.527
Parasutterella	0.000	0.000	0.000
Helicobacter	0.000	0.000	0.000
Akkermansia	0.000	0.000	0.001

Supplementary Table 9. Identified 727 fecal metabolites and their relative abundance (signal intensity) in donor and recipient *db/db* mice treated with either regular (Ctrl) or RM drinking water. The values were calculated from 6 experimental samples for each group and subjected to 1-way ANOVA analysis.

BIOCHEMICAL	PATHWAY	dRM/dCtrl (mean)	rRM/rCtrl (mean)	dRM vs dCtrl (p-value)	rRM vs rCtrl (p-value)
glycine	Amino Acid	1.49	1.27	0.001	0.037
N-acetylglycine	Amino Acid	0.2	0.86	0.000	0.861
sarcosine (N-Methylglycine)	Amino Acid	0.53	2.22	0.127	0.028
betaine	Amino Acid	0.62	0.64	0.218	0.246
serine	Amino Acid	1.4	1.2	0.023	0.200
N-acetylserine	Amino Acid	0.69	2.38	0.014	0.044
O-acetylserine	Amino Acid	1.19	4.22	0.808	0.001
threonine	Amino Acid	1.57	1.14	0.001	0.281
N-acetylthreonine	Amino Acid	0.39	1.56	0.002	0.146
homoserine	Amino Acid	0.97	0.6	0.381	0.158
alanine	Amino Acid	1.46	1.46	0.011	0.005
N-acetylalanine	Amino Acid	0.32	0.73	0.001	0.428
aspartate	Amino Acid	1.53	1.17	0.030	0.340
asparagine	Amino Acid	1.19	0.42	0.713	0.026
N-acetylasparagine	Amino Acid	0.43	0.74	0.003	0.599
N-acetylaspartate (NAA)	Amino Acid	0.44	1.65	0.000	0.016
glutamate	Amino Acid	0.87	0.79	0.327	0.177
glutamine	Amino Acid	1.1	1.25	0.537	0.131
N-acetylglutamate	Amino Acid	0.34	0.75	0.000	0.127
N-acetylglutamine	Amino Acid	0.2	0.42	0.007	0.068
gamma-aminobutyrate (GABA)	Amino Acid	1.41	0.97	0.326	0.809
carboxyethyl-GABA*	Amino Acid	1.09	0.73	0.632	0.049
glutamate, gamma-methyl ester	Amino Acid	1.11	1.03	0.650	0.729
pyroglutamine*	Amino Acid	0.41	0.63	0.004	0.055
N-methyl-4-aminobutyric acid	Amino Acid	0.67	3.31	0.053	0.000
histidine	Amino Acid	1.65	1.28	0.007	0.110
N-acetylhistidine	Amino Acid	0.52	2.29	0.006	0.006
N-acetyl-1-methylhistidine*	Amino Acid	0.07	0.25	0.050	0.342
trans-urocanate	Amino Acid	1.6	1.3	0.012	0.103
formiminoglutamate	Amino Acid	1.07	1.66	0.542	0.012
imidazole propionate	Amino Acid	1.06	1.4	0.712	0.387
imidazole lactate	Amino Acid	0.68	15.1	0.027	0.000
histamine	Amino Acid	0.18	0.96	0.000	0.809
1-methylimidazoleacetate	Amino Acid	0.22	0.67	0.020	0.203
4-imidazoleacetate	Amino Acid	0.29	1.23	0.027	0.629
lysine	Amino Acid	2.1	1.35	0.000	0.077
N2-acetyllysine	Amino Acid	0.74	0.93	0.019	0.575
N6-acetyllysine	Amino Acid	1.23	1.05	0.260	0.646
5-hydroxylysine	Amino Acid	1.58	0.23	0.136	0.000

saccharopine	Amino Acid	0.87	0.46	0.152	0.044
2-aminoadipate	Amino Acid	1.14	0.81	0.563	0.337
2-oxoadipate	Amino Acid	0.01	0.05	0.046	0.003
glutarate (pentanedioate)	Amino Acid	0.74	0.32	0.161	0.000
glutarylcarnitine (C5)	Amino Acid	0.26	0.23	0.177	0.003
3-methylglutarylcarnitine (1)	Amino Acid	0.2	0.28	0.049	0.023
pipecolate	Amino Acid	0.55	0.59	0.018	0.035
N-acetyl-cadaverine	Amino Acid	0.84	3.68	0.378	0.000
5-aminovalerate	Amino Acid	1.2	2.95	0.514	0.001
N6-carboxyethyllysine	Amino Acid	0.92	0.62	0.569	0.006
phenylalanine	Amino Acid	2.17	1.54	0.000	0.007
N-acetylphenylalanine	Amino Acid	1.15	1.95	0.565	0.004
phenylpyruvate	Amino Acid	0.47	0.27	0.049	0.001
phenyllactate (PLA)	Amino Acid	0.56	1.93	0.011	0.023
phenethylamine	Amino Acid	1.71	0.79	0.420	0.338
4-hydroxyphenylacetate	Amino Acid	0.84	1.39	0.225	0.107
phenylacetylglycine	Amino Acid	0.08	0.29	0.047	0.015
phenylacetylglutamine	Amino Acid	0.37	0.92	0.251	0.755
tyrosine	Amino Acid	2.11	1.58	0.000	0.009
N-acetyltyrosine	Amino Acid	0.92	3.24	0.457	0.000
4-hydroxycinnamate	Amino Acid	0.75	0.59	0.051	0.006
tyramine	Amino Acid	1.06	0.9	0.704	0.503
4-hydroxyphenylpyruvate	Amino Acid	0.39	0.21	0.007	0.000
3-(4-hydroxyphenyl)lactate	Amino Acid	0.76	1.01	0.131	0.966
phenol sulfate	Amino Acid	0.04	0.44	0.048	0.056
p-cresol sulfate	Amino Acid	0.04	0.19	0.049	0.007
dihydoxyphenylalanine (L-DOPA)	Amino Acid	2.44	3.08	0.029	0.003
3-methoxytyrosine	Amino Acid	0.57	0.66	0.315	0.269
dihydrocaffeate	Amino Acid	0.27	0.29	0.008	0.001
gentisate	Amino Acid	0.9	1.13	0.449	0.691
phenylpropionylglycine	Amino Acid	2.74	4.22	0.125	0.127
2-phenylpropionate	Amino Acid	90.92	176.69	0.000	0.000
3-[3-(sulfooxy)phenyl]propanoic acid	Amino Acid	0.01	0.03	0.001	0.000
2-(4-hydroxyphenyl)propionate	Amino Acid	0.72	0.27	0.130	0.000
3-(3-hydroxyphenyl)propionate	Amino Acid	0.17	0.27	0.000	0.000
3-(4-hydroxyphenyl)propionate	Amino Acid	0.09	0.13	0.000	0.000
3-phenylpropionate (hydrocinnamate)	Amino Acid	52.44	83.73	0.000	0.000
5-hydroxymethyl-2-furoic acid	Amino Acid	0.32	0.35	0.022	0.004
2-hydroxyphenylacetate	Amino Acid	0.16	0.23	0.143	0.012
tryptophan	Amino Acid	1.78	1.85	0.002	0.001
N-acetyltryptophan	Amino Acid	2.07	3.81	0.019	0.001
tryptamine	Amino Acid	1.34	2.16	0.390	0.002
indolelactate	Amino Acid	0.9	2.78	0.288	0.008
indoleacetate	Amino Acid	0.8	2.43	0.277	0.001
indolepropionate	Amino Acid	0.89	3.73	0.163	0.020
3-indoxyl sulfate	Amino Acid	0.03	0.52	0.043	0.051
kynurenine	Amino Acid	1.43	1.07	0.003	0.578

kynurenate	Amino Acid	0.62	0.44	0.526	0.013
anthranilate	Amino Acid	0.54	0.8	0.226	0.450
xanthurenate	Amino Acid	0.49	0.34	0.319	0.009
picolinate	Amino Acid	0.67	0.87	0.329	0.693
5-hydroxyindoleacetate	Amino Acid	0.28	0.38	0.012	0.007
serotonin (5HT)	Amino Acid	1.02	0.93	0.897	0.398
C-glycosyltryptophan*	Amino Acid	0.07	0.31	0.060	0.019
indole-3-carboxylic acid	Amino Acid	0.83	3.32	0.235	0.000
leucine	Amino Acid	2.47	1.81	0.000	0.000
N-acetylleucine	Amino Acid	0.26	0.63	0.034	0.630
4-methyl-2-oxopentanoate	Amino Acid	0.42	0.35	0.018	0.003
isovalerate	Amino Acid	1.51	2.37	0.208	0.003
isovalerylglycine	Amino Acid	0.11	0.21	0.024	0.010
isovalerylcarnitine	Amino Acid	0.5	0.46	0.428	0.106
beta-hydroxyisovalerate	Amino Acid	1.06	0.85	0.500	0.718
3-methylglutaconate	Amino Acid	0.25	0.32	0.056	0.018
alpha-hydroxyisovalerate	Amino Acid	0.79	2.34	0.049	0.006
methylsuccinate	Amino Acid	0.55	0.73	0.025	0.171
isoleucine	Amino Acid	2.44	1.82	0.000	0.000
allo-isoleucine	Amino Acid	0.95	1.16	0.349	0.903
N-acetylisoleucine	Amino Acid	0.35	1.69	0.159	0.079
3-methyl-2-oxovalerate	Amino Acid	0.43	0.35	0.027	0.003
2-methylbutyrylcarnitine (C5)	Amino Acid	0.77	0.61	0.406	0.083
2-methylbutyrylglycine	Amino Acid	0.86	1.54	0.383	0.108
2-hydroxy-3-methylvalerate	Amino Acid	0.73	3.44	0.047	0.001
3-hydroxy-2-ethylpropionate	Amino Acid	0.94	1.73	0.127	0.181
ethylmalonate	Amino Acid	0.4	0.43	0.177	0.102
valine	Amino Acid	2.14	1.62	0.000	0.003
N-acetylvaline	Amino Acid	0.63	3.78	0.457	0.001
3-methyl-2-oxobutyrate	Amino Acid	0.38	0.36	0.016	0.005
isobutyrylcarnitine	Amino Acid	0.47	1.16	0.015	0.745
isobutyrylglycine	Amino Acid	0.83	1.39	0.234	0.056
3-hydroxyisobutyrate	Amino Acid	0.61	0.54	0.062	0.016
alpha-hydroxyisocaproate	Amino Acid	0.77	2.24	0.047	0.012
methionine	Amino Acid	1.28	2.52	0.457	0.112
N-acetylmethionine	Amino Acid	1.11	3.52	0.803	0.000
N-formylmethionine	Amino Acid	0.89	2.61	0.832	0.002
methionine sulfone	Amino Acid	1.84	1.78	0.176	0.328
methionine sulfoxide	Amino Acid	1.44	2.41	0.070	0.001
N-acetylmethionine sulfoxide	Amino Acid	1.51	2.43	0.127	0.000
2-aminobutyrate	Amino Acid	2.37	2.26	0.004	0.004
2-hydroxybutyrate (AHB)	Amino Acid	0.79	2.09	0.125	0.014
cysteine	Amino Acid	1.93	2.11	0.013	0.002
N-acetylcysteine	Amino Acid	0.62	0.71	0.618	0.381
cystine	Amino Acid	2.84	1.7	0.113	0.109
cysteine s-sulfate	Amino Acid	1.64	1.8	0.041	0.011
hypotaurine	Amino Acid	0.26	0.56	0.013	0.234

taurine	Amino Acid	0.43	8.53	0.051	0.000
N-acetyltaurine	Amino Acid	0.71	1.04	0.065	0.878
arginine	Amino Acid	3.18	1.3	0.000	0.185
urea	Amino Acid	0.34	0.4	0.233	0.181
ornithine	Amino Acid	1.77	3.62	0.115	0.000
proline	Amino Acid	1.88	2.13	0.000	0.000
citrulline	Amino Acid	1.12	2.31	0.870	0.000
homocitrulline	Amino Acid	1.63	1.31	0.002	0.040
dimethylarginine (SDMA + ADMA)	Amino Acid	1.13	1.14	0.917	0.425
N-acetylarginine	Amino Acid	1.03	0.5	0.607	0.002
N-acetylproline	Amino Acid	0.93	0.94	0.862	0.818
N-delta-acetylornithine	Amino Acid	0.47	1.7	0.002	0.036
N2,N5-diacetylornithine	Amino Acid	0.19	0.49	0.001	0.081
N-alpha-acetylornithine	Amino Acid	0.91	1.16	0.511	0.518
N-methyl proline	Amino Acid	0.87	0.8	0.235	0.308
trans-4-hydroxyproline	Amino Acid	0.99	1.96	0.979	0.000
pro-hydroxy-pro	Amino Acid	2.51	2.65	0.173	0.104
creatine	Amino Acid	1.11	0.78	0.638	0.267
creatinine	Amino Acid	0.48	0.36	0.136	0.008
guanidinoacetate	Amino Acid	0.11	0.27	0.032	0.017
acisoga	Amino Acid	0.24	0.28	0.066	0.036
spermidine	Amino Acid	3.51	0.89	0.008	0.729
5-methylthioadenosine (MTA)	Amino Acid	4.15	1.01	0.005	0.884
N-acetylputrescine	Amino Acid	0.98	0.47	0.571	0.000
4-acetamidobutanoate	Amino Acid	0.49	0.6	0.021	0.035
4-guanidinobutanoate	Amino Acid	0.24	0.28	0.135	0.006
guanidinosuccinate	Amino Acid	0.2	0.64	0.094	0.228
5-oxoproline	Amino Acid	0.63	0.94	0.024	0.796
gamma-glutamylisoleucine*	Peptide	0.67	2.71	0.048	0.000
gamma-glutamylleucine	Peptide	1.83	3.24	0.020	0.000
gamma-glutamyllysine	Peptide	2.14	2.71	0.216	0.002
gamma-glutamylmethionine	Peptide	0.89	4.04	0.593	0.006
gamma-glutamylphenylalanine	Peptide	1.2	1.61	0.226	0.002
gamma-glutamyltryptophan	Peptide	1.6	3.31	0.283	0.004
gamma-glutamyltyrosine	Peptide	1.13	1.58	0.444	0.005
gamma-glutamylvaline	Peptide	1.74	2.66	0.019	0.000
carnosine	Peptide	0.79	0.67	0.635	0.139
anserine	Peptide	0.91	1.61	0.911	0.397
alanylalanine	Peptide	1.82	1.69	0.229	0.073
alanylglutamate	Peptide	1.7	1.67	0.006	0.012
alanylglutamine	Peptide	2.06	2.27	0.018	0.017
alanylisoleucine	Peptide	1.44	1.22	0.153	0.355
alanylleucine	Peptide	1.91	2.65	0.007	0.000
alanylmethionine	Peptide	2.15	2.37	0.002	0.000
alanylphenylalanine	Peptide	2.83	6.7	0.001	0.000
alanylproline	Peptide	1.16	0.5	0.942	0.409
alanyltyrosine	Peptide	3.14	14.19	0.001	0.000

alanylvaline	Peptide	1.35	1.24	0.177	0.305
alpha-glutamylglutamate	Peptide	1.22	0.75	0.836	0.449
alpha-glutamylthreonine	Peptide	3.97	2.02	0.000	0.005
alpha-glutamyltryptophan	Peptide	3.15	4.01	0.000	0.000
alpha-glutamyltyrosine	Peptide	2.58	3.47	0.000	0.000
alpha-glutamylvaline	Peptide	1.62	1.59	0.233	0.077
arginylisoleucine	Peptide	1.29	2.4	0.300	0.001
arginylleucine	Peptide	1.48	3.3	0.304	0.000
arginylphenylalanine	Peptide	1.96	5.55	0.137	0.000
arginylproline	Peptide	1.11	1	0.992	0.916
arginylvaline	Peptide	1.16	1.31	0.646	0.425
asparagylisoleucine	Peptide	1.36	1.31	0.165	0.169
asparagylleucine	Peptide	1.8	2.64	0.002	0.000
asparagylvaline	Peptide	1.52	1.43	0.022	0.036
aspartate-glutamate	Peptide	1.12	1.08	0.527	0.892
aspartylleucine	Peptide	2.21	1.9	0.000	0.001
aspartylphenylalanine	Peptide	2.06	2.52	0.000	0.000
aspartyltryptophan	Peptide	2.56	1.47	0.006	0.257
aspartylvaline	Peptide	1.97	1.3	0.002	0.076
cyclo(gly-glu)	Peptide	1.14	2.69	0.419	0.000
cyclo(gly-pro)	Peptide	0.42	0.49	0.014	0.014
cyclo(leu-phe)	Peptide	0.16	0.63	0.000	0.081
cyclo(leu-pro)	Peptide	0.56	1.69	0.182	0.156
cyclo(L-phe-L-pro)	Peptide	0.47	1.78	0.105	0.341
cyclo(phe-phe)	Peptide	0.37	1	0.001	1.000
cyclo(pro-tyr)	Peptide	0.92	0.77	0.281	0.003
glutamine-isoleucine	Peptide	1.48	1.38	0.088	0.152
glutamine-leucine	Peptide	1.85	2.29	0.003	0.000
glycylglycine	Peptide	1.72	1.05	0.000	0.794
glycylisoleucine	Peptide	1.52	0.91	0.085	0.895
glycylleucine	Peptide	1.83	1.19	0.002	0.303
glycylmethionine	Peptide	2.44	1.41	0.001	0.117
glycylphenylalanine	Peptide	2.7	2.43	0.000	0.000
glycylproline	Peptide	1.37	1	0.069	0.967
glycylserine	Peptide	1.87	1.16	0.000	0.342
glycyltryptophan	Peptide	2.7	1.53	0.000	0.067
glycyltyrosine	Peptide	2.21	2.49	0.001	0.000
glycylvaline	Peptide	1.56	1.01	0.055	0.873
histidylglycine	Peptide	4.76	1.51	0.000	0.124
histidylisoleucine	Peptide	3.59	1.53	0.005	0.097
histidylleucine	Peptide	3.4	2.1	0.000	0.002
histidylphenylalanine	Peptide	3.11	3.75	0.000	0.000
histidylproline	Peptide	1.29	1.18	0.581	0.699
histidyltryptophan	Peptide	4.51	1.39	0.002	0.341
histidyltyrosine	Peptide	6.77	6.09	0.000	0.001
histidylvaline	Peptide	1.91	1.21	0.012	0.232
isoleucylalanine	Peptide	1.87	1.64	0.028	0.071

isoleucylarginine	Peptide	5.9	1.84	0.000	0.114
isoleucylasparagine	Peptide	1.94	1.17	0.010	0.741
isoleucylaspartate	Peptide	1.15	1.03	0.394	0.938
isoleucylglutamate	Peptide	1.33	1.15	0.079	0.404
isoleucylglutamine	Peptide	1.9	1.85	0.005	0.005
isoleucylglycine	Peptide	1.99	1.19	0.000	0.278
isoleucylisoleucine	Peptide	1.47	1.23	0.155	0.359
isoleucylleucine	Peptide	1.71	2.3	0.015	0.001
isoleucylmethionine	Peptide	2.3	2.27	0.008	0.013
isoleucylphenylalanine	Peptide	2.57	3.67	0.001	0.000
isoleucylserine	Peptide	1.73	1.12	0.007	0.512
isoleucylthreonine	Peptide	1.25	1.12	0.197	0.533
isoleucyltryptophan	Peptide	2.35	1.55	0.001	0.093
isoleucyltyrosine	Peptide	3.1	4.68	0.000	0.000
isoleucylvaline	Peptide	1.32	1.04	0.243	0.787
leucylalanine	Peptide	1.98	1.29	0.035	0.465
leucylarginine	Peptide	4.83	1.33	0.001	0.550
leucylasparagine	Peptide	2.28	0.83	0.020	0.466
leucylglutamate	Peptide	1.11	0.83	0.417	0.182
leucylglycine	Peptide	1.56	1.03	0.009	0.913
leucylisoleucine	Peptide	1.4	1.71	0.227	0.112
leucylleucine	Peptide	1.97	2.08	0.021	0.033
leucylmethionine	Peptide	2.31	1.86	0.022	0.129
leucylphenylalanine	Peptide	2.99	4.96	0.002	0.000
leucylserine	Peptide	1.47	1.04	0.123	0.903
leucylthreonine	Peptide	1.25	0.96	0.234	0.899
leucyltryptophan	Peptide	2.85	2.1	0.003	0.067
leucyltyrosine	Peptide	3.55	3.77	0.001	0.001
lysylisoleucine	Peptide	1.89	1.87	0.027	0.016
lysylleucine	Peptide	1.78	3.04	0.040	0.000
lysylvaline	Peptide	1.56	1.5	0.077	0.060
methionylalanine	Peptide	2.14	1.68	0.028	0.121
methionylglutamate	Peptide	1.36	1.07	0.318	0.779
methionylphenylalanine	Peptide	4.61	6.43	0.000	0.000
methionylthreonine	Peptide	1.88	1.6	0.044	0.238
methionylvaline	Peptide	1.6	1.9	0.211	0.154
phenylalanylalanine	Peptide	2.61	1.34	0.011	0.470
phenylalanylarginine	Peptide	7.59	0.89	0.001	0.683
phenylalanylaspartate	Peptide	1.52	0.89	0.099	0.712
phenylalanylglutamate	Peptide	1.29	0.89	0.175	0.622
phenylalanylglycine	Peptide	1.86	1.1	0.007	0.693
phenylalanylisoleucine	Peptide	1.94	1.51	0.040	0.325
phenylalanylleucine	Peptide	2.74	2.53	0.003	0.015
phenylalanylphenylalanine	Peptide	3.48	6.4	0.000	0.000
phenylalanylproline	Peptide	0.82	0.81	0.345	0.256
phenylalanylserine	Peptide	1.7	1.09	0.017	0.661
phenylalanyltyrosine	Peptide	5.06	3.73	0.000	0.002

phenylalanylvaline	Peptide	1.6	1.21	0.073	0.606
prolylalanine	Peptide	1.51	1.23	0.012	0.235
prolylglutamine	Peptide	2.04	1.14	0.000	0.329
prolylglycine	Peptide	1.47	0.99	0.002	0.906
prolylproline	Peptide	1.62	1.83	0.025	0.005
prolyltryptophan	Peptide	3.69	3.52	0.002	0.002
prolylvaline	Peptide	1.64	1.54	0.046	0.047
pyroglutamylvaline	Peptide	1.1	0.95	0.298	0.654
serylisoleucine	Peptide	1.37	1.07	0.159	0.641
serylleucine	Peptide	1.56	1.75	0.017	0.004
serylmethionine	Peptide	1.79	1.72	0.017	0.027
serylphenyalanine	Peptide	2.13	3.51	0.001	0.000
seryltyrosine	Peptide	1.99	3.93	0.001	0.000
serylvaline	Peptide	1.34	1.12	0.118	0.511
threonylarginine	Peptide	2.84	1.16	0.000	0.660
threonylisoleucine	Peptide	1.16	1.15	0.503	0.442
threonylleucine	Peptide	1.95	2.95	0.002	0.000
threonylmethionine	Peptide	2.14	2.28	0.003	0.001
threonylphenylalanine	Peptide	2.53	4.68	0.000	0.000
threonylvaline	Peptide	1.29	1.11	0.292	0.524
tryptophylalanine	Peptide	3.28	2.15	0.007	0.127
tryptophylglutamate	Peptide	1.47	1.12	0.059	0.613
tryptophylglycine	Peptide	2.47	1.85	0.003	0.085
tryptophylleucine	Peptide	4.43	4.72	0.000	0.001
tryptophyltryptophan	Peptide	4.47	3.24	0.000	0.005
tryptophylvaline	Peptide	2.38	4.11	0.010	0.002
tyrosylalanine	Peptide	2.22	0.98	0.002	0.915
tyrosylglutamate	Peptide	1.6	0.93	0.027	0.805
tyrosylglycine	Peptide	2.37	1.52	0.003	0.103
tyrosylleucine	Peptide	3.03	1.76	0.003	0.129
tyrosyllysine	Peptide	9.81	1	0.001	0.749
tyrosylphenylalanine	Peptide	4.52	4.36	0.001	0.001
tyrosyltyrosine	Peptide	6.71	2.29	0.000	0.064
tyrosylvaline	Peptide	2.56	1.21	0.114	0.490
valylalanine	Peptide	1.61	1.48	0.039	0.093
valylarginine	Peptide	8.34	2.38	0.001	0.058
valylasparagine	Peptide	2.25	1.13	0.000	0.512
valylaspartate	Peptide	1.15	1.05	0.467	0.752
valylglutamate	Peptide	1.24	1.21	0.421	0.965
valylglycine	Peptide	1.77	1.19	0.000	0.199
valylisoleucine	Peptide	1.28	1.26	0.314	0.391
valylleucine	Peptide	1.88	1.88	0.005	0.006
valylmethionine	Peptide	2.32	2.02	0.004	0.015
valylphenylalanine	Peptide	2.33	3.45	0.001	0.000
valylthreonine	Peptide	1.55	1.1	0.019	0.553
valyltryptophan	Peptide	2.29	1.68	0.010	0.197
valyltyrosine	Peptide	2.82	2.97	0.000	0.000

valylvaline	Peptide	1.27	1.06	0.289	0.753
leucylglutamine*	Peptide	1.57	1.33	0.070	0.355
val-val-val	Peptide	0.8	1.39	0.299	0.140
glucose	Carbohydrate	0.51	0.32	0.014	0.000
glucose-6-phosphate (G6P)	Carbohydrate	3.01	0.67	0.797	0.829
pyruvate	Carbohydrate	0.7	0.5	0.044	0.000
lactate	Carbohydrate	0.74	1.97	0.083	0.039
glycerate	Carbohydrate	0.79	1.06	0.118	0.722
ribulose	Carbohydrate	1.44	1.28	0.557	0.428
ribose	Carbohydrate	0.67	0.96	0.008	0.690
ribitol	Carbohydrate	0.32	0.24	0.016	0.001
ribonate	Carbohydrate	0.66	2.76	0.233	0.001
xylose	Carbohydrate	0.9	0.92	0.475	0.572
arabinose	Carbohydrate	1.23	1.13	0.947	0.706
threitol	Carbohydrate	0.46	1.45	0.256	0.548
fucose	Carbohydrate	0.79	1.16	0.284	0.503
maltotriose	Carbohydrate	0.61	0.72	0.058	0.311
maltose	Carbohydrate	0.2	0.93	0.000	0.743
stachyose	Carbohydrate	0.31	1.73	0.000	0.009
lactobionate	Carbohydrate	0.87	1.99	0.146	0.018
sucrose	Carbohydrate	0.77	0.36	0.448	0.124
cellobiose	Carbohydrate	0.2	0.6	0.000	0.053
raffinose	Carbohydrate	0.34	0.45	0.062	0.448
fructose	Carbohydrate	0.47	0.58	0.022	0.077
sorbitol	Carbohydrate	0.57	0.45	0.226	0.229
mannose	Carbohydrate	0.86	0.22	0.199	0.000
mannitol	Carbohydrate	0.1	0.09	0.001	0.001
rhamnose	Carbohydrate	0.79	1.29	0.113	0.077
galactose	Carbohydrate	0.85	0.94	0.141	0.701
galactonate	Carbohydrate	0.77	1.32	0.103	0.191
glucosamine	Carbohydrate	0.39	0.34	0.002	0.000
glucuronate	Carbohydrate	0.49	0.59	0.012	0.025
N-acetylglucosamine	Carbohydrate	0.98	1.67	0.594	0.054
N-acetylglucosamine 6-sulfate	Carbohydrate	0.96	2.42	0.637	0.001
N-acetylgalactosamine	Carbohydrate	1.25	1.15	0.389	0.355
N-acetylneuraminate	Carbohydrate	1.09	1.17	0.818	0.327
N-acetylmuramate	Carbohydrate	0.6	2.13	0.021	0.005
erythronate*	Carbohydrate	0.85	0.75	0.590	0.288
N6-carboxymethyllysine	Carbohydrate	1.88	0.81	0.002	0.167
citrate	Energy	0.1	0.1	0.014	0.001
aconitate [cis or trans]	Energy	0.2	0.19	0.017	0.002
alpha-ketoglutarate	Energy	0.16	0.04	0.001	0.000
succinate	Energy	1.1	0.74	0.934	0.222
fumarate	Energy	0.62	0.64	0.092	0.255
malate	Energy	0.8	0.58	0.195	0.011
tricarballylate	Energy	0.78	0.47	0.189	0.093
phosphate	Energy	0.86	0.6	0.198	0.088

valerate	Lipid	2.77	4.34	0.152	0.006
heptanoate (7:0)	Lipid	1.34	2.01	0.387	0.005
caprylate (8:0)	Lipid	1.46	1.71	0.052	0.003
caprate (10:0)	Lipid	1.02	1.08	0.937	0.586
laurate (12:0)	Lipid	1.01	1.02	0.868	0.807
5-dodecenoate (12:1n7)	Lipid	1.03	1.36	0.831	0.049
myristate (14:0)	Lipid	0.85	0.9	0.207	0.404
myristoleate (14:1n5)	Lipid	1.03	1.36	0.723	0.002
pentadecanoate (15:0)	Lipid	0.77	0.87	0.306	0.576
palmitate (16:0)	Lipid	1	1.04	0.801	0.778
palmitoleate (16:1n7)	Lipid	0.98	1.27	0.629	0.029
margarate (17:0)	Lipid	1.27	0.93	0.639	0.853
10-heptadecenoate (17:1n7)	Lipid	1.28	1.36	0.106	0.028
stearate (18:0)	Lipid	1.01	0.87	0.947	0.939
oleate (18:1n9)	Lipid	0.63	0.99	0.001	0.936
cis-vaccenate (18:1n7)	Lipid	0.57	0.68	0.086	0.288
nonadecanoate (19:0)	Lipid	0.96	0.72	0.978	0.715
10-nonadecenoate (19:1n9)	Lipid	1.1	1.59	0.666	0.153
7-nonadecenoate (19:1n12)	Lipid	0.87	1.63	0.483	0.069
arachidate (20:0)	Lipid	0.83	0.92	0.626	0.810
eicosenoate (20:1n9 or 11)	Lipid	1.08	0.88	0.849	0.817
erucate (22:1n9)	Lipid	0.88	1.3	0.736	0.502
stearidonate (18:4n3)	Lipid	0.79	1.35	0.082	0.064
eicosapentaenoate (EPA; 20:5n3)	Lipid	1.16	1.51	0.391	0.005
docosapentaenoate (n3 DPA; 22:5n3)	Lipid	1.03	1.07	0.991	0.728
docosahexaenoate (DHA; 22:6n3)	Lipid	1.5	2.37	0.020	0.000
omega-3 arachidonate (20:4n3)	Lipid	0.87	0.98	0.331	0.918
linoleate (18:2n6)	Lipid	0.85	1.29	0.185	0.063
linolenate [alpha or gamma; (18:3n3 or 6)]	Lipid	0.8	1.18	0.035	0.156
dihomo-linolenate (20:3n3 or n6)	Lipid	1.13	0.98	0.522	0.926
arachidonate (20:4n6)	Lipid	1.73	2.11	0.006	0.000
adrenate (22:4n6)	Lipid	0.75	1.3	0.428	0.504
docosapentaenoate (n6 DPA; 22:5n6)	Lipid	1.73	1.59	0.099	0.146
docosadienoate (22:2n6)	Lipid	0.48	0.57	0.354	0.645
dihomo-linoleate (20:2n6)	Lipid	0.61	1.05	0.429	0.812
mead acid (20:3n9)	Lipid	1.96	1.67	0.045	0.150
12-methyltridecanoic acid	Lipid	0.78	0.66	0.132	0.019
13-methylmyristic acid	Lipid	1.3	0.91	0.249	0.676
15-methylpalmitate (isobar with 2-methylpalmit	Lipid	0.96	1.04	0.868	0.852
17-methylstearate	Lipid	0.93	0.79	0.818	0.855
dimethylmalonic acid	Lipid	0.49	1.07	0.035	0.714
2-hydroxyglutarate	Lipid	0.4	0.71	0.004	0.209
adipate	Lipid	0.61	0.53	0.183	0.038
2-hydroxyadipate	Lipid	0.45	0.72	0.119	0.172
maleate (cis-Butenedioate)	Lipid	1.01	1.54	0.898	0.001
suberate (octanedioate)	Lipid	0.92	0.73	0.300	0.003
azelate (nonanedioate)	Lipid	1.09	1.03	0.559	0.701

sebacate (decanedioate)	Lipid	1.08	1.62	0.675	0.001
undecanedioate	Lipid	0.88	1.21	0.312	0.123
dodecanedioate	Lipid	0.7	0.91	0.176	0.570
hexadecanedioate	Lipid	1.58	1.21	0.005	0.250
docosadioate	Lipid	0.92	0.79	0.777	0.177
linoleamide (18:2n6)	Lipid	1.68	0.34	0.580	0.391
2-aminoheptanoate	Lipid	1.18	0.95	0.323	0.808
malonate (propanedioate)	Lipid	0.72	1.15	0.050	0.388
2-methylmalonyl carnitine	Lipid	0.57	0.39	0.106	0.019
butyrylcarnitine	Lipid	0.44	0.37	0.048	0.041
butyrylglycine	Lipid	0.08	0.18	0.012	0.007
hexanoylglycine	Lipid	0.11	0.14	0.020	0.001
N-palmitoyl glycine	Lipid	1.64	1.06	0.233	0.837
acetylcarnitine	Lipid	0.33	0.71	0.343	0.172
hexanoylcarnitine	Lipid	0.71	0.55	0.170	0.125
palmitoylcarnitine	Lipid	1.66	1.08	0.301	0.891
stearoylcarnitine	Lipid	1.68	1	0.256	1.000
deoxycarnitine	Lipid	1.74	0.89	0.013	0.797
carnitine	Lipid	0.65	0.37	0.106	0.001
acetoacetate	Lipid	1.46	1.67	0.614	0.950
3-hydroxybutyrate (BHBA)	Lipid	2.63	1.76	0.501	0.428
4-hydroxybutyrate (GHB)	Lipid	3.39	0.71	0.011	0.356
alpha-hydroxycaproate	Lipid	0.77	1.17	0.321	0.538
2-hydroxypalmitate	Lipid	0.72	1.41	0.610	0.156
2-hydroxystearate	Lipid	0.61	0.88	0.455	0.867
3-hydroxyoctanoate	Lipid	1.04	1.32	0.778	0.144
3-hydroxysebacate	Lipid	0.95	0.85	0.623	0.435
3-hydroxypalmitate	Lipid	1.29	1.35	0.128	0.101
5-hydroxyhexanoate	Lipid	0.71	0.94	0.147	0.840
16-hydroxypalmitate	Lipid	1.15	1.41	0.774	0.174
13-HODE + 9-HODE	Lipid	0.72	1.05	0.060	0.803
10-hydroxystearate	Lipid	1.05	0.36	0.566	0.000
12,13-DiHOME	Lipid	1.39	0.88	0.009	0.228
9,10-DiHOME	Lipid	1.72	1.11	0.069	0.783
12(13)-EpOME	Lipid	1.16	0.88	0.491	0.349
9(10)-EpOME	Lipid	0.81	0.99	0.093	0.978
oleic ethanolamide	Lipid	0.91	0.78	0.973	0.408
palmitoyl ethanolamide	Lipid	0.92	0.52	0.927	0.010
N-oleoyltaurine	Lipid	1.15	7.22	0.887	0.027
N-stearoyltaurine	Lipid	1.14	3.37	0.983	0.090
N-palmitoyltaurine	Lipid	0.61	2.07	0.885	0.271
myo-inositol	Lipid	1.23	0.67	0.629	0.053
chiro-inositol	Lipid	0.6	0.8	0.072	0.610
pinitol	Lipid	0.44	0.29	0.053	0.022
inositol 1-phosphate (I1P)	Lipid	0.76	0.58	0.524	0.120
choline	Lipid	0.91	1.03	0.419	0.852
choline phosphate	Lipid	0.73	0.62	0.311	0.134

glycerophosphorylcholine (GPC)	Lipid	1.32	1.21	0.178	0.323
ethanolamine	Lipid	0.96	0.49	0.732	0.010
glycerophosphoethanolamine	Lipid	1.09	0.83	0.702	0.289
glycerophosphoinositol*	Lipid	0.64	0.57	0.002	0.000
1-palmitoylglycerophosphocholine (16:0)	Lipid	0.77	2.62	0.633	0.003
2-palmitoylglycerophosphocholine*	Lipid	0.75	1.76	0.668	0.106
1-margaroylglycerophosphocholine (17:0)	Lipid	0.99	2.74	0.958	0.003
1-stearoylglycerophosphocholine (18:0)	Lipid	0.9	2.99	0.945	0.003
1-oleoylglycerophosphocholine (18:1)	Lipid	0.67	2.1	0.558	0.023
2-oleoylglycerophosphocholine*	Lipid	1.22	0.59	0.561	0.645
1-linoleoylglycerophosphocholine (18:2n6)	Lipid	0.65	2.61	0.356	0.002
2-linoleoylglycerophosphocholine*	Lipid	1.01	0.28	0.698	0.001
2-arachidonoylglycerophosphocholine*	Lipid	1.71	0.62	0.060	0.342
1-palmitoylplasmenylethanolamine*	Lipid	1.55	0.57	0.372	0.996
1-stearoylplasmenylethanolamine*	Lipid	1.02	0.92	0.909	0.845
1-oleoylplasmenylethanolamine*	Lipid	1.95	0.87	0.225	0.465
1-myristoylglycerophosphoethanolamine	Lipid	0.82	2.06	0.534	0.013
1-palmitoylglycerophosphoethanolamine	Lipid	0.69	1.55	0.250	0.060
2-palmitoylglycerophosphoethanolamine*	Lipid	0.58	1.26	0.348	0.317
1-stearoylglycerophosphoethanolamine	Lipid	0.83	2.55	0.557	0.003
2-stearoylglycerophosphoethanolamine*	Lipid	0.73	2.95	0.327	0.032
1-oleoylglycerophosphoethanolamine	Lipid	1.02	1.26	0.880	0.173
2-oleoylglycerophosphoethanolamine*	Lipid	1.09	0.78	0.685	0.473
1-linoleoylglycerophosphoethanolamine*	Lipid	0.91	1.25	0.750	0.338
2-linoleoylglycerophosphoethanolamine*	Lipid	0.98	0.4	0.993	0.268
2-arachidonoylglycerophosphoethanolamine*	Lipid	1.22	0.56	0.619	0.505
1-palmitoylglycerophosphoinositol*	Lipid	0.63	2.79	0.105	0.002
1-stearoylglycerophosphoinositol	Lipid	0.84	3.8	0.372	0.001
1-oleoylglycerophosphoinositol*	Lipid	1.11	0.33	0.967	0.059
1-linoleoylglycerophosphoinositol*	Lipid	1.82	0.26	0.364	0.014
1-stearoylglycerophosphoserine*	Lipid	0.85	2.33	0.640	0.006
1-palmitoylglycerophosphate	Lipid	2.91	1.35	0.795	0.485
1-palmitoylglycerophosphoglycerol*	Lipid	2.26	1.33	0.407	0.399
2-palmitoylglycerophosphoglycerol*	Lipid	1.47	0.52	0.761	0.198
1-stearoylglycerophosphoglycerol	Lipid	2.45	2.6	0.406	0.017
2-stearoylglycerophosphoglycerol*	Lipid	3.08	0.45	0.493	0.373
1-oleoylglycerophosphoglycerol*	Lipid	1.79	5.87	0.613	0.002
2-oleoylglycerophosphoglycerol*	Lipid	2.29	1.84	0.323	0.284
palmitoyl-linoleoyl-glycerophosphoinositol (1)*	Lipid	0.49	2.57	0.009	0.000
glycerol	Lipid	0.7	1.15	0.001	0.116
glycerol 3-phosphate (G3P)	Lipid	5.74	2.48	0.145	0.127
1-myristoylglycerol (1-monomyristin)	Lipid	0.93	1.14	0.858	0.431
2-myristoylglycerol (2-monomyristin)	Lipid	0.99	0.94	0.764	0.983
1-pentadecanoylglycerol (1-monopentadecano	Lipid	1.22	1.15	0.644	0.203
1-palmitoylglycerol (1-monopalmitin)	Lipid	0.72	0.85	0.090	0.470
2-palmitoylglycerol (2-monopalmitin)	Lipid	0.99	0.97	0.940	0.915
1-stearoylglycerol (1-monostearin)	Lipid	0.96	1.31	0.898	0.106

2-stearoylglycerol (2-monostearin)	Lipid	1.07	1.64	0.740	0.017
1-oleoylglycerol (1-monoolein)	Lipid	1.03	1.13	0.872	0.592
2-oleoylglycerol (2-monoolein)	Lipid	1.01	0.94	0.841	0.887
1-linoleoylglycerol (1-monolinolein)	Lipid	0.97	0.85	0.896	0.978
2-linoleoylglycerol (2-monolinolein)	Lipid	0.9	0.39	0.943	0.217
1-linolenoylglycerol	Lipid	0.82	0.93	0.739	0.815
1-docosahexaenoylglycerol (1-monodocosahe:	Lipid	0.77	1.32	0.300	0.265
1-dihomo-linolenylglycerol (alpha, gamma)	Lipid	0.61	1.3	0.344	0.734
3-ketosphinganine	Lipid	1.21	2.11	0.689	0.019
sphinganine	Lipid	0.79	1.05	0.506	0.528
phytosphingosine	Lipid	1.57	2.57	0.541	0.012
sphingosine	Lipid	0.86	1.85	0.746	0.040
3-hydroxy-3-methylglutarate	Lipid	0.93	0.31	0.974	0.003
mevalonate	Lipid	0.52	0.67	0.003	0.047
mevalonolactone	Lipid	0.44	0.83	0.002	0.425
squalene	Lipid	0.8	0.91	0.890	0.816
cholesterol	Lipid	0.78	0.86	0.069	0.341
cholestanol	Lipid	0.57	1.25	0.004	0.232
coprostanol	Lipid	0.1	0.96	0.000	0.773
campesterol	Lipid	1.04	0.73	0.938	0.149
campestanol	Lipid	0.67	1.18	0.215	0.638
cholate	Lipid	1.59	12.75	0.148	0.014
glycocholate	Lipid	1.53	7.86	0.398	0.000
taurocholate	Lipid	0.36	1.89	0.030	0.151
chenodeoxycholate	Lipid	1.4	5.47	0.651	0.001
taurochenodeoxycholate	Lipid	0.38	2.92	0.004	0.001
beta-muricholate	Lipid	0.92	6.17	0.960	0.000
alpha-muricholate	Lipid	0.54	8.88	0.336	0.000
tauro-alpha-muricholate	Lipid	0.3	6.15	0.005	0.000
tauro-beta-muricholate	Lipid	0.34	5.05	0.009	0.000
deoxycholate	Lipid	0.28	4.92	0.000	0.000
taurodeoxycholate	Lipid	0.03	6.62	0.000	0.001
6-beta-hydroxylithocholate	Lipid	0.78	4.63	0.511	0.000
lithocholate	Lipid	0.24	2.43	0.000	0.001
taurolithocholate	Lipid	0.05	4.54	0.000	0.001
taurolithocholate 3-sulfate	Lipid	0.04	1.38	0.000	0.556
ursodeoxycholate	Lipid	1.34	7.94	0.429	0.000
tauroursodeoxycholate	Lipid	0.63	3.39	0.075	0.001
dehydrolithocholate	Lipid	0.22	2.3	0.000	0.018
7,12-diketolithocholate	Lipid	2.01	26.38	0.078	0.000
6-oxolithocholate	Lipid	0.47	1.36	0.010	0.383
7-ketolithocholate	Lipid	0.55	15.47	0.565	0.000
hyocholate	Lipid	1.6	2.22	0.114	0.016
taurohyodeoxycholic acid	Lipid	0.25	2.31	0.000	0.009
3-dehydrocholate	Lipid	3.75	17.04	0.006	0.004
12-dehydrocholate	Lipid	3.1	26.01	0.013	0.001
taurocholenate sulfate	Lipid	0.27	17.94	0.015	0.000

7-ketodeoxycholate	Lipid	0.75	14.89	0.545	0.002
3b-hydroxy-5-cholenoic acid	Lipid	2.17	2.54	0.052	0.057
inosine	Nucleotide	6.19	1.09	0.079	0.971
hypoxanthine	Nucleotide	1.23	1.06	0.684	0.938
xanthine	Nucleotide	0.91	0.63	0.435	0.001
xanthosine	Nucleotide	3.08	1.01	0.125	0.738
2'-deoxyinosine	Nucleotide	0.84	0.92	0.515	0.614
urate	Nucleotide	0.48	0.25	0.084	0.002
allantoin	Nucleotide	0.1	0.36	0.058	0.033
allantoic acid	Nucleotide	0.03	0.56	0.108	0.203
adenosine 5'-monophosphate (AMP)	Nucleotide	0.73	0.12	0.314	0.000
adenosine 3'-monophosphate (3'-AMP)	Nucleotide	0.98	1.15	0.601	0.579
adenosine 3',5'-cyclic monophosphate (cAMP)	Nucleotide	1	0.25	1.000	0.045
adenosine	Nucleotide	0.38	1.78	0.000	0.057
adenine	Nucleotide	0.3	1.19	0.000	0.824
1-methyladenine	Nucleotide	0.17	0.32	0.277	0.019
N1-methyladenosine	Nucleotide	1.25	0.74	0.757	0.499
N6-methyladenosine	Nucleotide	1.65	0.59	0.292	0.135
N6,N6-dimethyladenosine	Nucleotide	2.66	0.34	0.095	0.006
N6-carbamoylthreonyladenosine	Nucleotide	1	0.33	1.000	0.024
2'-deoxyadenosine 5'-monophosphate	Nucleotide	0.43	0.09	0.389	0.023
2'-deoxyadenosine	Nucleotide	0.24	1.71	0.000	0.569
N6-succinyladenosine	Nucleotide	0.47	0.35	0.058	0.048
guanosine-2',3'-cyclic monophosphate	Nucleotide	1.21	1.44	0.684	0.594
guanosine	Nucleotide	3.52	1.2	0.036	0.683
guanine	Nucleotide	0.94	1.54	0.588	0.005
7-methylguanine	Nucleotide	1.14	0.78	0.502	0.447
N1-methylguanosine	Nucleotide	1.7	0.94	0.150	0.816
N2,N2-dimethylguanosine	Nucleotide	1	0.58	1.000	0.037
N2,N2-dimethylguanine	Nucleotide	0.23	0.24	0.050	0.005
2'-deoxyguanosine	Nucleotide	0.77	0.82	0.598	0.526
N-carbamoylaspartate	Nucleotide	0.27	0.9	0.059	0.899
orotate	Nucleotide	0.45	0.52	0.021	0.023
uridine 5'-monophosphate (UMP)	Nucleotide	0.59	1.06	0.143	0.439
uridine-3'-monophosphate (3'-UMP)	Nucleotide	0.7	2.77	0.658	0.094
uridine	Nucleotide	1.36	0.88	0.228	0.431
uracil	Nucleotide	1.01	1.03	0.755	0.981
pseudouridine	Nucleotide	0.58	0.4	0.270	0.023
5-methyluridine (ribothymidine)	Nucleotide	1.53	0.71	0.494	0.197
N3-methyluridine	Nucleotide	0.95	0.6	0.941	0.038
5,6-dihydrouracil	Nucleotide	0.74	0.33	0.427	0.025
2'-deoxyuridine	Nucleotide	0.97	0.72	0.780	0.078
3-ureidopropionate	Nucleotide	0.16	0.21	0.107	0.030
beta-alanine	Nucleotide	1.19	1.13	0.744	0.786
N-acetyl-beta-alanine	Nucleotide	0.88	0.93	0.369	0.805
cytidine 5'-monophosphate (5'-CMP)	Nucleotide	0.96	0.23	0.822	0.000
cytidine-3'-monophosphate (3'-CMP)	Nucleotide	1.14	0.93	0.898	0.861

cytidine	Nucleotide	1.17	1.11	0.789	0.701
cytosine	Nucleotide	0.34	1.9	0.000	0.070
N4-acetylcytidine	Nucleotide	1	2.01	1.000	0.001
2'-deoxycytidine 5'-monophosphate	Nucleotide	0.57	0.29	0.033	0.000
2'-deoxycytidine	Nucleotide	0.73	0.93	0.295	0.674
5-methyl-2'-deoxycytidine	Nucleotide	1.11	1.01	0.650	0.823
thymidine 5'-monophosphate	Nucleotide	0.24	0.21	0.002	0.002
thymidine	Nucleotide	0.94	0.82	0.951	0.399
thymine	Nucleotide	0.86	0.9	0.436	0.486
5,6-dihydrothymine	Nucleotide	1.01	0.61	0.764	0.418
methylphosphate	Nucleotide	0.93	0.48	0.801	0.036
nicotinate	Cofactors and Vitamins	1.07	1.34	0.958	0.101
nicotinate ribonucleoside	Cofactors and Vitamins	2.28	2.14	0.314	0.101
nicotinamide	Cofactors and Vitamins	0.76	1.27	0.407	0.980
nicotinamide riboside	Cofactors and Vitamins	4.06	2.14	0.006	0.038
nicotinamide adenine dinucleotide (NAD+)	Cofactors and Vitamins	1.32	1.45	0.812	0.296
1-methylnicotinamide	Cofactors and Vitamins	0.08	0.62	0.074	0.407
6-hydroxynicotinate	Cofactors and Vitamins	2.17	1.71	0.409	0.090
trigonelline (N'-methylnicotinate)	Cofactors and Vitamins	0.43	0.43	0.013	0.005
N1-Methyl-2-pyridone-5-carboxamide	Cofactors and Vitamins	0.12	0.52	0.275	0.123
riboflavin (Vitamin B2)	Cofactors and Vitamins	0.82	1.04	0.247	0.993
flavin adenine dinucleotide (FAD)	Cofactors and Vitamins	1.2	0.7	0.571	0.705
flavin mononucleotide (FMN)	Cofactors and Vitamins	1.22	1.4	0.840	0.519
pantothenate	Cofactors and Vitamins	0.75	1.61	0.065	0.025
threonate	Cofactors and Vitamins	0.56	0.63	0.141	0.172
arabonate	Cofactors and Vitamins	0.98	4.05	0.870	0.000
gulonic acid*	Cofactors and Vitamins	0.08	0.2	0.015	0.017
alpha-tocopherol	Cofactors and Vitamins	0.92	1.04	0.829	0.990
gamma-tocopherol	Cofactors and Vitamins	0.6	0.66	0.056	0.056
biotin	Cofactors and Vitamins	0.84	1.01	0.230	0.948
biopterin	Cofactors and Vitamins	0.19	0.37	0.172	0.076
dihydrobiopterin	Cofactors and Vitamins	0.76	0.84	0.161	0.290
pterin	Cofactors and Vitamins	0.67	1	0.090	0.725
biliverdin	Cofactors and Vitamins	1.61	0.62	0.206	0.349
I-urobilinogen	Cofactors and Vitamins	3.85	3.11	0.013	0.070
D-urobilin	Cofactors and Vitamins	2.09	2.04	0.000	0.000
L-urobilin	Cofactors and Vitamins	0.99	1.42	0.996	0.140
pyridoxine (Vitamin B6)	Cofactors and Vitamins	0.72	2.41	0.481	0.008
pyridoxal	Cofactors and Vitamins	0.68	0.91	0.000	0.306
pyridoxate	Cofactors and Vitamins	0.86	0.95	0.116	0.648
hippurate	Xenobiotics	1.04	2.21	0.532	0.997
2-hydroxyhippurate (salicylurate)	Xenobiotics	0.08	0.13	0.033	0.002
3-hydroxyhippurate	Xenobiotics	0.15	0.13	0.007	0.000
4-hydroxyhippurate	Xenobiotics	0.02	0.12	0.002	0.004
benzoate	Xenobiotics	1	1.43	0.932	0.175
4-hydroxybenzoate	Xenobiotics	0.55	1.11	0.107	0.663
2,4,6-trihydroxybenzoate	Xenobiotics	0.92	2.08	0.338	0.000

catechol sulfate	Xenobiotics	0.02	0.14	0.065	0.014
4-methylcatechol sulfate	Xenobiotics	0.02	0.09	0.055	0.005
p-aminobenzoate (PABA)	Xenobiotics	0.8	0.58	0.059	0.000
4-vinylphenol sulfate	Xenobiotics	0.2	0.08	0.229	0.008
1-methylurate	Xenobiotics	1.59	2.07	0.308	0.047
1,3-dimethylurate	Xenobiotics	0.74	9.97	0.316	0.000
1-methylxanthine	Xenobiotics	2.52	0.37	0.023	0.004
maltol	Xenobiotics	0.61	3.6	0.071	0.000
2-piperidinone	Xenobiotics	0.86	1.03	0.187	0.723
kaempferol	Xenobiotics	0.77	3.03	0.396	0.001
genistein	Xenobiotics	1.11	4.36	0.620	0.000
1-methyl-beta-carboline-3-carboxylic acid	Xenobiotics	1.04	0.74	0.745	0.060
diaminopimelate	Xenobiotics	1.35	7.11	0.323	0.000
apigenin	Xenobiotics	0.63	1.67	0.085	0.032
luteolin	Xenobiotics	1	1.06	0.954	0.790
levulinate (4-oxovalerate)	Xenobiotics	1.27	1.13	0.272	0.676
vanillate	Xenobiotics	0.97	0.67	0.505	0.024
1H-quinolin-2-one	Xenobiotics	0.95	5.05	0.389	0.000
2,3-dihydroxyisovalerate	Xenobiotics	1.2	1.96	0.345	0.010
2,8-quinolinediol	Xenobiotics	0.22	89.35	0.000	0.000
2-isopropylmalate	Xenobiotics	0.62	0.28	0.277	0.000
2-oxindole-3-acetate	Xenobiotics	1.06	0.63	0.545	0.005
3,5-dihydroxybenzoic acid	Xenobiotics	0.05	0.93	0.000	0.908
3-hydroxyindolin-2-one	Xenobiotics	0.15	0.91	0.013	0.487
gluconate	Xenobiotics	0.67	1.18	0.079	0.940
5-ketogluconate	Xenobiotics	0.44	0.46	0.036	0.009
6-hydroxydaidzein	Xenobiotics	0.59	6.2	0.037	0.000
biochanin A	Xenobiotics	0.97	1.69	0.919	0.392
caffeate	Xenobiotics	1.29	0.67	0.202	0.054
chrysoeriol	Xenobiotics	0.75	2.26	0.175	0.002
cinnamoylglycine	Xenobiotics	5.42	7.71	0.025	0.132
daidzein	Xenobiotics	0.81	1.68	0.267	0.101
daidzin	Xenobiotics	1.15	0.78	0.849	0.257
dihydroferulic acid	Xenobiotics	1.3	2.16	0.383	0.069
enterolactone	Xenobiotics	0.76	0.69	0.030	0.005
equol	Xenobiotics	0.86	0.98	0.058	0.724
equol glucuronide	Xenobiotics	1.31	0.26	0.692	0.055
equol sulfate	Xenobiotics	0.12	0.24	0.045	0.018
ergothioneine	Xenobiotics	0.67	5.17	0.019	0.001
eriodictyol	Xenobiotics	0.58	1.76	0.062	0.097
erythritol	Xenobiotics	0.38	0.38	0.139	0.036
ferulate	Xenobiotics	0.97	0.93	0.700	0.654
ferulic acid 4-sulfate	Xenobiotics	0.14	0.24	0.033	0.008
formononetin	Xenobiotics	0.88	1.35	0.875	0.562
fucitol	Xenobiotics	1.35	0.76	0.195	0.129
galacturonate	Xenobiotics	0.39	0.97	0.002	0.879
glycitein	Xenobiotics	1.45	1.14	0.388	0.939

homostachydrine*	Xenobiotics	0.31	0.4	0.038	0.055
indolin-2-one	Xenobiotics	0.13	2.9	0.000	0.012
isoferulate	Xenobiotics	0.76	0.93	0.205	0.998
methyl indole-3-acetate	Xenobiotics	0.56	0.6	0.272	0.194
N-(2-furoyl)glycine	Xenobiotics	0.05	0.2	0.012	0.015
naringenin	Xenobiotics	0.53	1.58	0.030	0.094
N-glycolylneuraminate	Xenobiotics	0.47	0.29	0.222	0.007
nicotianamine	Xenobiotics	3.91	18.85	0.135	0.000
oleanolate	Xenobiotics	0.95	0.99	0.643	0.900
pheophorbide A	Xenobiotics	1.29	1.13	0.143	0.495
quinate	Xenobiotics	1.08	1	0.619	0.880
sinapate	Xenobiotics	1.07	0.84	0.991	0.272
sitostanol	Xenobiotics	0.69	0.94	0.024	0.604
stachydrine	Xenobiotics	0.36	0.36	0.051	0.013
syringic acid	Xenobiotics	1.42	0.6	0.365	0.026
methyl glucopyranoside (alpha + beta)	Xenobiotics	0.64	0.29	0.220	0.000
enterodiol	Xenobiotics	0.89	1	0.306	0.972
5-sulfosalicylate	Xenobiotics	0.1	0.07	0.049	0.001
6-oxopiperidine-2-carboxylic acid	Xenobiotics	0.77	0.6	0.297	0.028
hydroquinone sulfate	Xenobiotics	0.04	0.09	0.033	0.001
salicylate	Xenobiotics	0.44	0.72	0.016	0.309
1,2-propanediol	Xenobiotics	1.03	1.22	0.947	0.973
sulfate*	Xenobiotics	0.21	0.31	0.021	0.017
2-oxo-1-pyrrolidinepropionate	Xenobiotics	0.29	0.58	0.004	0.043
2-aminophenol sulfate	Xenobiotics	0.02	0.18	0.053	0.010
S-(3-hydroxypropyl)mercapturic acid (HPMA)	Xenobiotics	0.16	0.29	0.184	0.032
dimethyl sulfone	Xenobiotics	0.2	0.26	0.043	0.032
ectoine	Xenobiotics	0.36	0.38	0.000	0.000
glycolate (hydroxyacetate)	Xenobiotics	0.97	1.03	0.939	0.978
N-methylpipecolate	Xenobiotics	0.57	0.19	0.000	0.000

Supplementary Table 10. The metabolomic signature of Ctrl and RM fecal samples (both donors and recipients). The fold change and ANOVA p values are duplicated from Supplementary Table 9. Mann-Whitney test was also conducted for these metabolites and the p values are shown to be highly significant.

BIOCHEMICAL	PATHWAY	dRM/dCtrl (Fold change)	rRM/rCtrl (Fold change)	dRM vs dCtrl (ANOVA p-value)	rRM vs rCtrl (ANOVA p-value)	dRM vs dCtrl (MW Test p-value)	rRM vs rCtrl (MW Test p-value)
alpha-glutamyltryptophan	Peptide	3.15	4.01	0.000	0.000	0.004	0.002
3-(3-hydroxyphenyl)propionate	Amino Acid	0.17	0.27	0.000	0.000	0.004	0.002
3-(4-hydroxyphenyl)propionate	Amino Acid	0.09	0.13	0.000	0.000	0.004	0.002
2-phenylpropionate	Amino Acid	90.92	176.69	0.000	0.000	0.008	0.005
3-phenylpropionate	Amino Acid	52.44	83.73	0.000	0.000	0.004	0.002
aspartylphenylalanine	Peptide	2.06	2.52	0.000	0.000	0.004	0.002
threonylphenylalanine	Peptide	2.53	4.68	0.000	0.000	0.004	0.002
histidylphenylalanine	Peptide	3.11	3.75	0.000	0.000	0.004	0.002
alpha-glutamyltyrosine	Peptide	2.58	3.47	0.000	0.000	0.004	0.002
serylphenyalanine	Peptide	2.13	3.51	0.001	0.000	0.004	0.002
threonylleucine	Peptide	1.95	2.95	0.002	0.000	0.004	0.002
isoleucine	Amino Acid	2.44	1.82	0.000	0.000	0.004	0.009
valyltyrosine	Peptide	2.82	2.97	0.000	0.000	0.004	0.002
alanylphenylalanine	Peptide	2.83	6.7	0.001	0.000	0.004	0.002
cyclo(leu-phe)	Peptide	0.16	0.63	0.000	0.081	0.005	0.010
proline	Amino Acid	1.88	2.13	0.000	0.000	0.009	0.002
valylphenylalanine	Peptide	2.33	3.45	0.001	0.000	0.004	0.002
glycyltyrosine	Peptide	2.21	2.49	0.001	0.000	0.004	0.009
N-acetyltryptophan	Amino Acid	2.07	3.81	0.019	0.001	0.030	0.002
isoleucyltyrosine	Peptide	3.1	4.68	0.000	0.000	0.004	0.002
glycylphenylalanine	Peptide	2.7	2.43	0.000	0.000	0.004	0.015
D-urobilin	Cofactors and Vitam	2.09	2.04	0.000	0.000	0.017	0.002
tryptophan	Amino Acid	1.78	1.85	0.002	0.001	0.004	0.015
leucine	Amino Acid	2.47	1.81	0.000	0.000	0.004	0.037
glutamine-leucine	Peptide	1.85	2.29	0.003	0.000	0.004	0.004
N-methylpipecolate	Xenobiotics	0.57	0.19	0.000	0.000	0.009	0.002
seryltyrosine	Peptide	1.99	3.93	0.001	0.000	0.004	0.002
asparagylleucine	Peptide	1.8	2.64	0.002	0.000	0.017	0.002
methionylphenylalanine	Peptide	4.61	6.43	0.000	0.000	0.004	0.002
tryptophylleucine	Peptide	4.43	4.72	0.000	0.001	0.004	0.004
